# Role of Treg cell subsets in cardiovascular disease pathogenesis and potential therapeutic targets

**DOI:** 10.3389/fimmu.2024.1331609

**Published:** 2024-03-15

**Authors:** Yuanliang Xia, Di Gao, Xu Wang, Bin Liu, Xue Shan, Yunpeng Sun, Dashi Ma

**Affiliations:** Department of Cardiac Surgery, The First Hospital of Jilin University, Changchun, China

**Keywords:** cardiovascular disease, Treg cell, immunotherapy, immune microenvironment, atherosclerosis, hypertension

## Abstract

In the genesis and progression of cardiovascular diseases involving both innate and adaptive immune responses, inflammation plays a pivotal and dual role. Studies in experimental animals indicate that certain immune responses are protective, while others exacerbate the disease. T-helper (Th) 1 cell immune responses are recognized as key drivers of inflammatory progression in cardiovascular diseases. Consequently, the CD4+CD25+FOXP3+ regulatory T cells (Tregs) are gaining increasing attention for their roles in inflammation and immune regulation. Given the critical role of Tregs in maintaining immune-inflammatory balance and homeostasis, abnormalities in their generation or function might lead to aberrant immune responses, thereby initiating pathological changes. Numerous preclinical studies and clinical trials have unveiled the central role of Tregs in cardiovascular diseases, such as atherosclerosis. Here, we review the roles and mechanisms of Treg subsets in cardiovascular conditions like atherosclerosis, hypertension, myocardial infarction and remodeling, myocarditis, dilated cardiomyopathy, and heart failure. While the precise molecular mechanisms of Tregs in cardiac protection remain elusive, therapeutic strategies targeting Tregs present a promising new direction for the prevention and treatment of cardiovascular diseases.

## 1 Introduction

Cardiovascular diseases (CVD) stand as the leading global cause of mortality (1, 2). Despite receiving state-of-the-art preventative medical interventions, patients with CVD still face a significant risk of recurrent events. Much of this residual risk is attributed to immune-inflammatory responses (2). T-cell mediated inflammatory reactions have been identified as central in the pathogenesis of CVD (3, 4). Consequently, targeting infiltrating T-cell subsets might offer innovative and promising therapeutic strategies for CVD (5).

A specific subset of infiltrating T cells, the Treg cells, constitute approximately 5-10% of all peripheral CD4+ T cells and play pivotal roles in maintaining homeostasis, immunological balance, and tolerance (6, 7). Aberrant accumulation or functional anomalies of Treg cells are closely associated with autoimmune diseases, chronic inflammation, infection progression, and tumor development. CD4+CD25+Foxp3+ regulatory T (Treg) cells evolve from immature T cells activated by antigens and cytokines (8, 9). While primarily maturing in the thymus, they can also transdifferentiate from peripheral naive CD4+ T cells (10). The forkhead/winged-helix transcription factor (FOXP3) serves as a hallmark for Treg cells, and the regulatory T cells expressing the transcription factor Foxp3 belong to a predominantly suppressive T-cell lineage of dual origin (11, 12). A deficiency in FOXP3 might lead to Treg cell dysfunction (13, 14). The strategies Treg cells employ to regulate T and B cell responses remain intricate and largely elusive. However, certain experiments and studies suggest that Treg cells, with their anti-inflammatory properties, might counteract the development of CVD (15, 16).

Adaptive and innate immune responses exhibit a dual role in CVD. While some immune reactions provide protective effects during the early stages of the disease, others can turn detrimental when rendered ineffective (17). Given this backdrop, the immune system emerges as an enticing target for pioneering CVD preventive therapies (18, 19). By selectively modulating this immune response related to CVD, it’s plausible to devise novel treatments for the disease (20). The reduction or functional impairment of regulatory T cells (Tregs) may lead to an increase in the activity of pro-inflammatory immune cells, such as Th1 and Th17 cells, thereby enhancing the inflammatory response of the cardiovascular system. In the context of atherosclerosis, dysfunction of Tregs may exacerbate inflammation and endothelial dysfunction, accelerating the formation and progression of plaques (21). After myocardial infarction, the reduction in Tregs can lead to excessive cardiac inflammation, affecting heart repair and functional recovery. The decline in Tregs may also promote the process of cardiac fibrosis, especially in the context of certain cardiomyopathies (22). However, oxidative stress may lead to dysfunction of Tregs, particularly in environments related to cardiovascular diseases. High cholesterol levels can also affect the function and survival of Tregs. The possibility of selectively modulating protective and deleterious immune reactions in CVD may aid in a more personalized prevention and treatment regimen. For instance, LDL (low-density lipoprotein) accumulation in arterial walls is a key autoantigen in atherosclerosis (23, 24). Studies aiming to validate this concept by immunizing experimental animals with oxidized LDL particles inadvertently triggered atherogenic immunity involving regulatory T cells (25, 26).

In this review, we delve deep into the roles of Treg cells in immune modulation, their underlying mechanisms, and further investigate their impacts on atherosclerosis, myocarditis, and other cardiovascular diseases. Moreover, we spotlight and discuss experimental and clinical data on the potentiality of crafting immunotherapies to reduce cardiovascular risk. We will also recapitulate ongoing clinical studies and deliberate challenges linked to the development of effective and safe vaccines for CVD.

## 2 The pathogenesis of Treg cells in cardiovascular diseases

**Figure 1:**
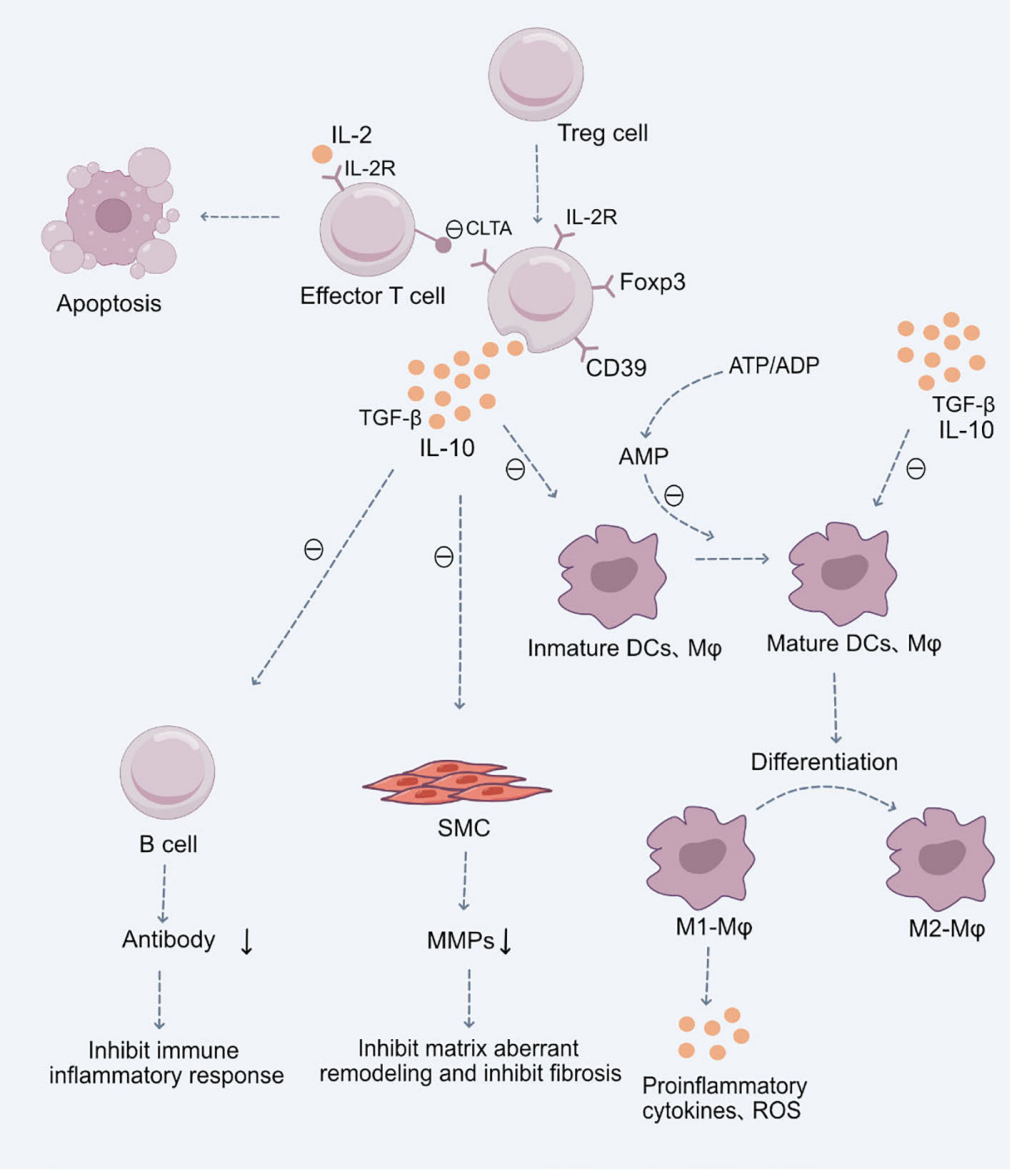
Role of Treg Cells in the Pathogenesis of Cardiovascular Diseases.Upon stimulation by antigens and cytokines, Treg cells differentiate into CD4+CD25+Foxp3+ Treg cells. These cells exert their immunosuppressive effects through the production of anti-inflammatory cytokines TGF-β and IL-10, subsequently inhibiting the functions of mature immune cells, including dendritic cells (DCs), macrophages, and effector T cells. Additionally, they suppress B cell antibody production, mitigating the immune-inflammatory response. They also inhibit the secretion of Matrix Metalloproteinases (MMPs) by smooth muscle cells (SMCs), restraining abnormal cardiovascular matrix remodeling and fibrosis. Moreover, CD4+CD25+Foxp3+ Treg cells facilitate the differentiation of pro-inflammatory M1 macrophages into anti-inflammatory M2 macrophages, thereby suppressing the production of inflammatory cytokines and reactive oxygen species (ROS). These Treg cells, with a high expression of the IL-2 receptor, competitively utilize IL-2, hindering the maturation of responding T cells and promoting their suppression and apoptosis. Certain inhibitory receptors expressed on Treg cells, such as CTLA-4, interact with ligands on antigen-presenting cells, inhibiting the latter’s function and inducing their apoptosis. Lastly, the ectoenzyme CD39 expressed by Treg cells hydrolyzes ATP or ADP to AMP, reinforcing the Treg-mediated suppression of ATP-driven dendritic cell maturation.

One of the primary mechanisms by which regulatory T cells (Tregs) participate in cardiovascular diseases is by modulating the immune-inflammatory responses of target T cells and antigen-presenting cells (APCs). This modulation limits T cell proliferation and cytokine production (27, 28). Tregs can regulate these immune-inflammatory responses through the release of inhibitory cytokines, the consumption of IL-2 and ATP/ADP, or through receptor-ligand interactions, such as inducing apoptosis or altering the functionality of APCs (29, 30) (**Figure 1**).

### 2.1 Production of anti-inflammatory cytokines by Tregs

Tregs play a pivotal role in the immune system by producing anti-inflammatory cytokines, such as TGF-β and IL-10. These cytokines can directly suppress other immune cells, including antigen-presenting cells (APCs) like macrophages and CD8+ effector T cells, thereby reducing inflammation and preventing excessive immune responses (31, 32).

Studies have highlighted the critical importance of TGF-β within Tregs *in vivo*, as Tregs in mice with a T-cell-specific deficiency of TGF-β failed to suppress inflammation (33, 34). Moreover, evidence suggests that Tregs can transmit membrane-bound TGF-β to corresponding receptors on effector T cells via direct cell-to-cell contact, thereby suppressing their functionality ex *vivo (* 35, 36).

Similarly, IL-10 has a crucial role in Treg-mediated immune modulation, especially in response to pathogens or external stimuli-induced inflammatory reactions (37, 38).IL-10 produced by Tregs plays a key role in resisting atherosclerosis and modulating the formation of atherosclerotic plaques (39). IL-10 can prevent endothelial cell dysfunction and help maintain the health of vascular endothelial cells, protecting them from damage by inflammatory factors (40). Additionally, it reduces the expression of adhesion molecules on endothelial cells: IL-10 can decrease the expression of adhesion molecules on the surface of endothelial cells, thereby reducing the adhesion and migration of inflammatory cells (32). IL-35, another cytokine predominantly expressed in Tregs, also has a significant role in the maximal immunoregulatory functions of Tregs (41, 42).IL-35 released by Tregs promotes the differentiation of naive Tregs into mature Tregs, while mice lacking IL-35 exhibited diminished Treg suppressive functions (41). Therefore, IL-35 is also a potential target for targeting Treg cells to inhibit immune-inflammatory damage.

### 2.2 Depletion of IL-2 by Treg cells stimulates pro-inflammatory cytokines

IL-2 is a pivotal growth factor, crucial for T cell proliferation. Although IL-2 itself does not directly produce anti-inflammatory effects, by promoting the activity of Tregs, IL-2 can indirectly promote the formation of an anti-inflammatory environment. The increase in Tregs helps regulate the immune response within atherosclerotic plaques, potentially aiding in reducing plaque formation and progression (43). Furthermore, by supporting the survival of Tregs, IL-2 helps maintain the balance of the immune system and immune tolerance, preventing excessive immune responses. This can help regulate the immune response within atherosclerotic plaques, potentially aiding in reducing plaque formation and progression (44). regulatory (Treg) cells possess high-affinity IL-2 receptors. By efficiently consuming IL-2, Treg cells “deplete” IL-2 from their surroundings, consequently inhibiting their own proliferation. This indirectly suppresses T cell-mediated inflammation (45, 46). IL-2 plays a critical role in the balance and development between Treg cells and effector T cells. Immune suppressive regulatory T lymphocytes expressing the transcription factor Foxp3 play an essential role in maintaining immune tolerance to self and benign non-self antigens (47). For most Tregs, differentiation requires antigenic signals from T cell receptors, costimulatory molecules, and signals from cytokine receptors like IL-2 (48, 49). Thus, by competitively utilizing IL-2, Treg cells interfere with the maturation of responsive T cells, leading to T cell apoptosis and suppression (47, 50).

Treg cells can absorb ATP and release ADP. The depletion and conversion of ATP/ADP can induce cytotoxicity and suppress the activities of nearby antigen-presenting cells (APCs) and CD8+ effector T cells (51, 52). The ectoenzyme CD39 expressed by Treg cells can hydrolyze ATP or ADP to AMP, enhancing Treg suppression of ATP-driven dendritic cell maturation (53, 54). Additionally, the co-expression of CD39 and CD73 on Tregs can convert extracellular ADP to adenosine. Adenosine can further bind to its A2A receptor, thereby inhibiting effector T cell activities (55, 56). Notably, activation of the adenosine A2A receptor not only suppresses effector T cells but also enhances Treg function by downregulating IL-6 expression and increasing TGF-β production (57, 58).

### 2.3 Treg cells mediate contact inhibition of antigen-presenting cells

Certain inhibitory receptors expressed on Treg cells, such as CTLA-4, can interact with ligands on antigen-presenting cells, inhibiting their function (59, 60).

Through regulation of antigen-presenting cells, particularly dendritic cells, and macrophages, Treg cells indirectly restrict effector T cell activity (61, 62). Tregs can inhibit dendritic cell function and maturation and induce them to produce TGF-β, further suppressing effector T cell activation and differentiation (63, 64). Studies have demonstrated that, in both humans and mice, Tregs can reduce the expression of the co-stimulatory molecules CD80 and CD86 on dendritic cells (63, 65). Highly expressed cytotoxic T lymphocyte-associated antigen-4 (CTLA-4) in Tregs plays a crucial role in this process, enhancing the regulation of dendritic cells. Some studies further indicate that Foxp3-expressing CD4^+CD25^+ regulatory T cells (Tregs) that highly express the immune checkpoint receptor CTLA-4 exhibit defects specific to Tregs, leading to severe immune inflammatory responses (63) As a critical mechanism of Treg-mediated suppression, CTLA-4 expressed by Tregs downregulates the expression of CD80/CD86 co-stimulatory molecules on antigen-presenting cells (APCs) (66, 67). Inflammation is a key factor leading to plaque instability and rupture; therefore, CTLA-4-mediated immune regulation may help increase plaque stability (68). Reducing inflammatory responses can protect vascular endothelium, preventing endothelial cell dysfunction, which is a key factor in the development of atherosclerosis and cardiovascular diseases. CTLA-4-mediated immune regulation helps control chronic inflammation, thereby aiding in the prevention of atherosclerosis progression and the occurrence of cardiovascular events (69).

Another molecule expressed in Treg cells that impacts dendritic cell function is lymphocyte-activation gene 3 (LAG3). It has a high affinity to class II molecules of the major histocompatibility complex (MHC) (70, 71). When LAG3 binds to MHC class II molecules, it activates the immunoreceptor tyrosine-based inhibitory signaling pathway, thereby reducing dendritic cell maturation and their capacity to activate T cells (72, 73).

### 2.4 Cytotoxic role in T-cell suppression

In T-cell suppression, cytotoxicity stands as a pivotal potential mechanism. For instance, CD8+ T cells and NK cells can directly target and kill cells through the Granzyme B and Perforin pathways (74, 75). Studies have identified that Treg cells lacking Granzyme B exhibit weakened suppressive functions. Granzyme B also plays a crucial role in the suppression of immunity by Treg cells, as it aids in the killing of NK cells and CD8+ T cells (76, 77). Granzyme B can induce apoptosis in cells within atherosclerotic plaques, such as macrophages and smooth muscle cells. An increase in cell apoptosis may lead to an increase in the cellular mass at the plaque core, affecting plaque stability. Imbalance in cell apoptosis may cause plaques to rupture more easily, thereby increasing the risk of acute cardiovascular events (76). Granzyme B-induced apoptosis of smooth muscle cells may affect the remodeling process of blood vessels, potentially impacting vascular stability and elasticity.

### 2.5 Treg cell-mediated suppression of B cells

Treg cells’ modulation of B cell responses showcases their vital impact. Experimental evidence suggests that when Treg cells are depleted, autoantibody production increases in autoimmune mice; conversely, supplementation of Treg cells results in decreased autoantibody concentrations (78, 79). An early hypothesis postulated that Treg cells primarily modulate B cell responses by suppressing the helper T cells that assist in antibody production (80, 81). However, subsequent research indicates that Treg cells might inhibit B cells’ class-switch recombination and induce apoptosis using perforin and granzymes, directly constraining antibody production (82, 83). B cells can produce pro-inflammatory antibodies, such as antibodies against oxidized low-density lipoprotein (oxLDL). These antibodies may promote inflammatory responses and the formation of atherosclerotic plaques. By inhibiting these B cells or reducing the pro-inflammatory antibodies they produce, inflammation and the progression of atherosclerosis can be mitigated (84).

### 2.6 Treg cells and endothelial cell interaction

Endothelial dysfunction is a key factor in the development of various cardiovascular diseases. Treg cells, by secreting anti-inflammatory cytokines such as TGF-β, can promote the integrity of the endothelial layer of blood vessels. Endothelial cells, through their membrane-bound TGFβ, convert some CD8(+) T cell populations into Treg cells. Treg cells induced by endothelial cells produce the soluble form of TGFβ1, but not TGFβ2, and they also acquire a regulatory phenotype expressing high levels of CD25 and Foxp3 (85). Vascular Endothelial Growth Factor A (VEGF-A), Interleukin 10 (IL-10), and Prostaglandin E2 (PGE2) synergistically induce the expression of FasL in endothelial cells. Due to the high expression levels of c-FLIP in Treg cells, they acquire the ability to kill effector CD8(+) T cells rather than Treg cells (86). Treg cells can reduce the expression of adhesion molecules on the surface of endothelial cells, decreasing the interaction between leukocytes and endothelial cells, thereby reducing vascular inflammation (87). Furthermore, studies have shown that the recognition of self-antigens expressed by endothelial cells in target tissues helps in the effective recruitment of Treg cells *in vivo*. This Treg recruitment depends on the induction of MHC class II molecule expression in endothelial cells mediated by IFN-γ, and requires the activation of the T cell receptor PI3K p110δ (88). Therefore, endothelial cells and self-recognition enable the transportation of Treg cells, expanding the understanding of immune regulation dynamics, and making the interaction between vascular endothelial cells and Treg cells a potential therapeutic target for treating cardiovascular diseases.

In summary, Treg cells maintain immune homeostasis through various mechanisms, ensuring that immune responses do not exceed the necessary thresholds. Within the context of cardiovascular diseases, Treg cell function might be compromised or insufficient, leading to intensified inflammatory responses, which can accelerate the progression of ailments like atherosclerosis. As such, modulating the function and number of Treg cells might emerge as a promising strategy in treating cardiovascular diseases.

## 3 Pathogenic mechanisms of Treg cells in cardiovascular diseases and potential therapeutic targets:

**Figure 2:**
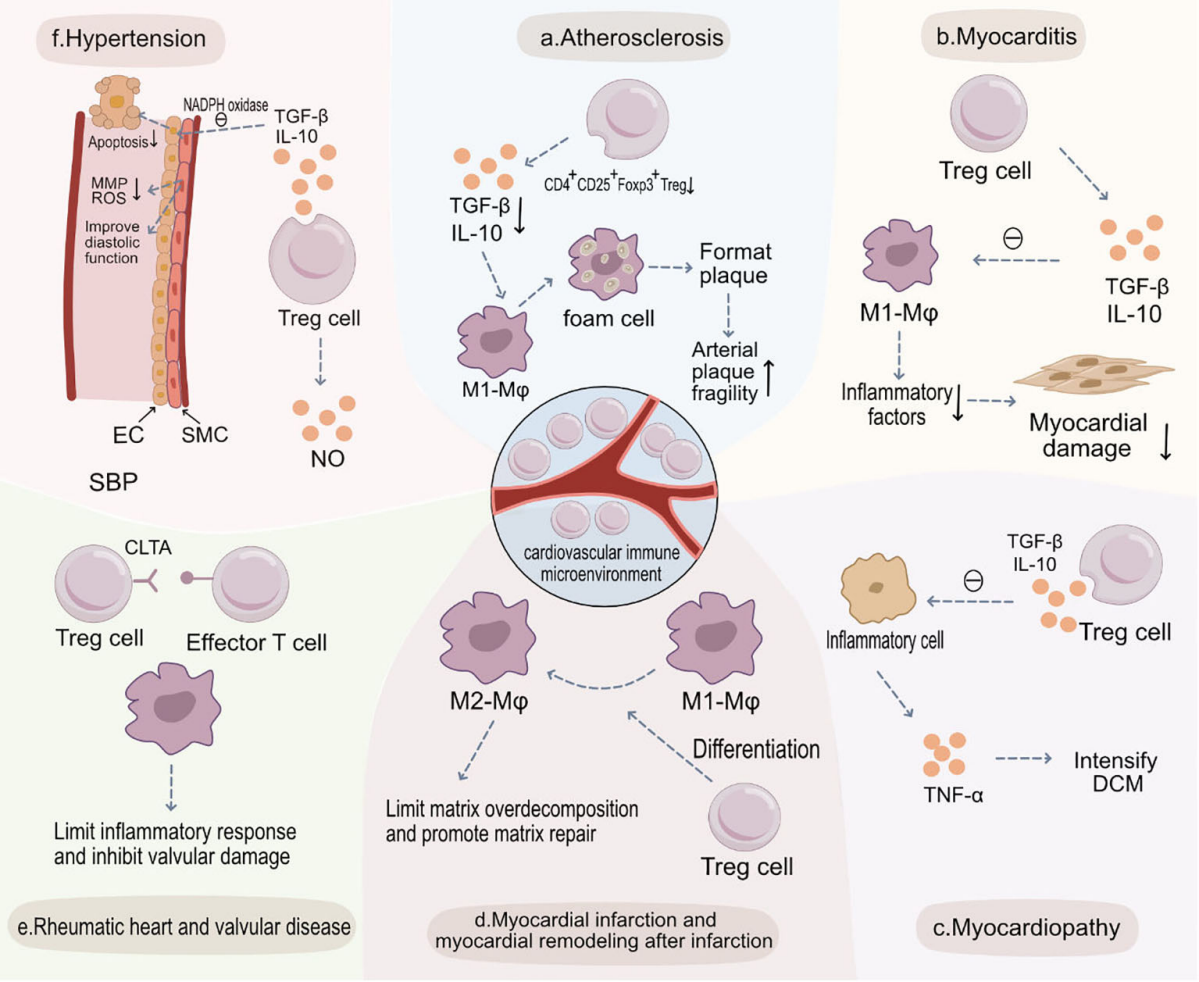
Treg Cells in the Pathogenesis of Various Cardiovascular Diseases and Potential Therapeutic Targets. **(A)** Atherosclerosis: Within atherosclerotic plaques, the abundance of CD4+CD25+Foxp3+ Treg cells diminishes, leading to a decreased release of anti-inflammatory cytokines such as TGF-β and IL-10. This results in increased engulfment of LDL by M1 macrophages and heightened foam cell formation, thus increasing plaque vulnerability. **(B)** Myocarditis: In myocarditis, CD4+CD25+Foxp3+ Treg cells release anti-inflammatory cytokines like TGF-β and IL-10, inhibiting the function of M1 macrophages, curbing immune-inflammatory reactions, and preserving myocardial tissue. **(C)** Myocardiopathy: In cardiomyopathies, there’s a decrease in CD4+CD25+Foxp3+ Treg cells and secretion levels of TGF-β and IL-10, weakening their inhibitory effects on inflammatory cells. This culminates in augmented secretion of pro-inflammatory cytokines like TNF-α, exacerbating dilated cardiomyopathy (DCM). **(D)** Myocardial Infarction and Post-Infarction Remodeling: Post-myocardial infarction, CD4+CD25+Foxp3+ Treg cells aid recovery by modulating the differentiation of monocytes and macrophages into reparative M2 macrophages. They also limit post-infarction inflammation and excessive matrix degradation, thereby slowing adverse morphological changes. **(E)** Rheumatic Heart and Valvular Disease: In rheumatic heart and valvular diseases, CD4+CD25+Foxp3+ Treg cells inhibit effector T cells and curtail sustained inflammatory responses, reducing valvular injury. **(F)** Hypertension: CD4+CD25+Foxp3+ Treg cells, through the secretion of TGF-β and IL-10, inhibit NADPH oxidase, restoring the vasodilatory function of SMCs and decreasing endothelial cell apoptosis to preserve endothelial functionality. Additionally, they suppress the secretion of MMPs and ROS by SMCs, preventing abnormal vascular remodeling and ultimately reducing systolic blood pressure (SBP).

Inflammation plays a key role in the onset and exacerbation of cardiovascular diseases (89). When inflammatory cells aggregate and release inflammatory cytokines, the progression of diseases like atherosclerosis, hypertension, and myocardial infarction can be accelerated (90, 91). Regulatory T cells (Tregs) play a pivotal role in controlling and suppressing inflammatory reactions, preserving the balance of the immune system, and preventing excessive immune responses (31, 92). A significant enhancement in inflammatory reactions is observed in the absence of Treg cells, hinting at the crucial role of Treg cells in maintaining cardiovascular health and suggesting their functionality might be influenced by their surrounding environment (93, 94). Treg cells can inhibit the aggregation of pro-inflammatory cells and the subsequent release of associated inflammatory cytokines, critical processes in cardiovascular diseases such as atherosclerosis (95). Therefore, amplifying or enhancing the function of Treg cells could present new strategies for treating cardiovascular diseases. Recognizing the role of Treg cells in cardiovascular ailments, researchers and clinicians are increasingly focusing on their potential as therapeutic targets. Modulating the number or function of Treg cells might offer novel approaches for preventing or treating inflammation-associated cardiovascular diseases (**Figure 2**).

### 3.1 Atherosclerosis

T regulatory cells (Tregs) play an indispensable role in the prevention and prognosis of atherosclerosis (96, 97). The role of regulatory T cells (Tregs) in atherosclerosis is highly diverse, including anti-inflammatory effects, maintaining immune balance, protecting vascular endothelium, and potential metabolic regulatory functions. Recent studies indicate that Tregs may also be involved in regulating metabolic pathways, such as lipid metabolism, which could significantly impact the progression of atherosclerosis (98). The ApoE-/- mouse model is extensively used in atherosclerosis research. Due to the absence of Apolipoprotein E (ApoE), these mice are prone to develop hypercholesterolemia and atherosclerosis (99, 100). Compared to normal mice, ApoE-/- mice exhibit a significant reduction in the number of Tregs (101, 102). Researchers have observed a reduced abundance of CD4+CD25+FOXP3+ Tregs in coronary artery atherosclerotic plaques, and this decrease correlates positively with the vulnerability of carotid artery plaques (103, 104) Tregs help protect vascular endothelial cells by reducing inflammation, thus preventing endothelial dysfunction. By decreasing the infiltration and activation of inflammatory cells, Tregs contribute to the stability of atherosclerotic plaques, reducing the risk of plaque rupture (105). further highlighting the protective role of Tregs further highlighting the protective role of Tregs.

Tregs are known to attenuate the accumulation of inflammatory cells, inhibit the secretion of inflammatory cytokines, and promote the transition of M1 macrophages to M2 phenotype. Consequently, this leads to a marked decrease in inflammatory cytokines and foam cells in atherosclerotic lesions (106). Anti-inflammatory cytokines released by Tregs, such as TGF-β, IL-10, and IL-35, not only enhance plaque stability but are also key in inhibiting atherosclerosis.

For instance, in the context of atherosclerosis, TGF-β exerts anti-inflammatory effects by inhibiting inflammatory cell activity, thereby preventing plaque formation and progression. Additionally, collagen, a crucial component for plaque stability, can degrade, increasing plaque vulnerability. TGF-β positively regulates collagen synthesis and deposition, aiding in maintaining vascular wall integrity (107, 108). Similarly, IL-10 also offers protection against atherosclerosis development. Absence of IL-10 exacerbates infiltration of inflammatory cells, reduces collagen content, and renders plaques more fragile. Overexpression of IL-10, on the other hand, counters these inflammatory responses and plaque formation (109, 110). In a study, induction of CD4+Foxp3+ Tregs in the spleens and aortas of ApoE-/- mice was frequently associated with significant elevations in plasma IL-35 levels (41). Further observations noted that CCR5+ Tregs in ApoE-/- exhibited a diminished AKT-mTOR signaling, elevated expression of inhibitory checkpoint receptors TIGIT and PD-1, enhanced TIGIT and PD-1 signaling, and increased IL-10 expression, all aiding in retaining the Treg immunosuppressive function (41). Therefore, IL-35 promotes the induction and differentiation of CD4+Foxp3+ Tregs, and by sustaining the suppressive mechanisms of CCR5 expanded Tregs, inhibits atherosclerosis.

B cells, pivotal cells in the immune system, primarily produce antibodies. A decrease in B cells in both ApoE-/- and LDLR-/- mice is associated with halting the progression of atherosclerosis, providing evidence of a probable pro-inflammatory role of B cells in atherosclerosis (111, 112). The exacerbation of the disease upon reintroduction of B cells further bolsters this perspective. Tregs are believed to suppress B cell activation, a suppression potentially mediated by cytokines produced by Tregs, such as IL-10 or TGF-β, or through direct cell-cell interactions (113, 114). Considering the regulatory effects of Tregs on B cells, enhancing the function or number of Tregs might also be an effective therapeutic strategy (115, 116). While current evidence has elucidated the critical roles of B cells and Tregs in atherosclerosis, more experiments are necessary to comprehensively understand their precise roles in the disease and how to best harness this information for new therapeutic approaches.

### 3.2 Myocarditis and cardiomyopathy

Myocarditis, characterized by inflammation, is a cardiac disease. Following viral myocarditis, autoimmune responses may lead to sustained myocardial damage. This could be pivotal in the transition of viral myocarditis to dilated cardiomyopathy (DCM) (117, 118). When Coxsackievirus B3 (CVB3) infects cardiomyocytes, it directly damages these cells, resulting in cell death and tissue injury, an effect attributed to its direct cytotoxicity (119, 120). Moreover, the body’s immune response targets the infecting virus; however, this response can occasionally be “overactive,” exacerbating myocardial damage. This phenomenon is referred to as post-viral pathogenic immune responses (121, 122). While the precise etiology of myocarditis remains to be fully elucidated, current studies speculate that autoimmune responses play a crucial role in disease onset and progression.

#### 3.2.1 Myocarditis and the role of Treg cells

Treg cells occupy a central protective role in the development of myocarditis (123).In animal models, experimental findings consistently show that natural Treg cells play a proactive role in suppressing virus-induced immunopathological responses and in preventing virus-induced tissue damage. Studies have identified a negative correlation between Treg cell abundance and the severity of myocarditis (124, 125). Thus far, numerous investigations have highlighted the critical protective function of Treg cells in myocarditis. Prior to viral infection, Treg cells can inhibit myocarditis induced by CVB3 by suppressing pathogenic immune responses and ensuring anti-viral cardiac responses via the TGF-β-Coxsackie adenovirus receptor pathway (125, 126). Tregs suppress cardiac inflammation by secreting anti-inflammatory cytokines, such as IL-10 and TGF-β. In the context of myocarditis, this suppression helps to reduce damage to cardiac tissues (32). Additionally, the suppressive functions of Treg cells have been displayed in multiple studies, suggesting that enhancing Treg cell activity can effectively alleviate myocarditis inflammation, reduce cardiac damage, and positively influence the progression of cardiomyopathy (127, 128).

However, the relationship between immune mechanisms and cardiomyopathy may not be linear or singular. Some findings regarding the role of Treg cells in myocarditis are contradictory. Tregs maintain the balance of the immune system by inhibiting excessive immune responses, which is particularly important in preventing the development of autoimmune cardiomyopathy. In the EAM (experimental autoimmune myocarditis) model, reducing Treg cells indeed exacerbates myocarditis, consistent with traditional understandings of Treg cells (129). If Treg cells suppress the immune response against the virus, they might indirectly promote viral replication and persistence, indicating a complex negative feedback mechanism (126). For instance, in EAM model studies, reducing the number of Treg cells within the heart resulted in aggravated myocarditis in mice (130). This seems to suggest that Treg cell activation might inhibit anti-viral immune responses, facilitating viral replication and persistence, further worsening myocardial changes. Such complexity underscores the need for a holistic perspective when understanding disease mechanisms. Within the context of myocarditis and DCM, it is essential to consider not just the interactions between the virus and the host but also the interplay among various cells within the immune system.

#### 3.2.2 Cardiomyopathy

Dilated cardiomyopathy (DCM) is a prevalent myocardial disorder characterized by ventricular dilation and diminished myocardial contractile function, which can potentially lead to heart failure (131, 132). In dilated cardiomyopathy, a major pathological feature is myocardial fibrosis. Tregs may help slow the progression of myocardial fibrosis by reducing the release of pro-fibrotic factors and inhibiting inflammation (133). Viral infections can instigate inflammatory and autoimmune responses in the myocardium, and persistent inflammation might lead to DCM (131, 134).

The TNF mouse model offers a tool to explore the relationship between immune mechanisms and DCM. TNF-α is a pro-inflammatory cytokine expressed in many inflammatory diseases (135). In the TNF mouse model, there is a heightened cardiac expression of TNF-α, leading to DCM characteristics like cardiac inflammation, ventricular dilation, and reduced ejection fraction (136, 137).

These findings suggest a potential link between immune mechanisms and the etiology and progression of DCM. Tregs may protect cardiac myocytes from damage by reducing inflammation and oxidative stress. Further research might unveil more about this disease’s mechanisms and provide insights for novel therapeutic strategies. Tregs may also play a role in the neovascularization and repair of the heart, which is crucial for the recovery and functional restoration of cardiomyopathies (125). Regulatory T (Treg) cells might play a role in inhibiting the progression of myocardial inflammation in DCM (125, 138). Moreover, when CD4+T cells are depleted in mice, their myocarditis symptoms are alleviated, further confirming the pivotal role of immune mechanisms in DCM pathogenesis (139, 140).

Compared to healthy individuals, DCM patients exhibit a decline in the number and function of Treg cells. Furthermore, serum levels of TGF-β and IL-10, both associated with immune suppression, are also reduced in DCM patients. This suggests that increasing the number and enhancing the function of circulating Treg cells might be therapeutic strategies for DCM (141, 142).

In conclusion, these experimental results provide insights into the intricate relationship between dilated cardiomyopathy and immune mechanisms. Modulating immune responses, especially by enhancing the function of Treg cells, could be a promising strategy for DCM treatment. However, translating these findings to clinical applications necessitates further research. The role of Tregs in myocarditis and cardiomyopathy is vital, primarily through immune regulation, anti-fibrotic effects, protecting cardiac myocytes and vascular functions, and regulating specific immune responses. These findings offer a new perspective and potential strategies for treating heart diseases, especially in treatments targeting the immune system (138).

### 3.3 Myocardial infarction and post-infarction cardiac remodeling

During myocardial ischemia and subsequent myocardial infarction (MI), immune responses play a crucial role in both injury and repair (15). Cell death induced by ischemia and infarction triggers an acute inflammatory response, drawing various immune cells to the injured myocardium (143). After myocardial infarction, the heart experiences an acute inflammatory response, and Tregs alleviate inflammation-mediated damage by secreting anti-inflammatory cytokines such as IL-10 and TGF-β (144). Tregs help control the immune response following myocardial infarction, preventing additional damage caused by excessive inflammation (64). Treg cells play a pivotal role in myocardial ischemia, MI, and post-infarction cardiac remodeling (16, 145).

Activated Treg cells soon after myocardial injury, due to their anti-inflammatory properties, maintain immune-inflammatory homeostasis, aiding tissue repair. In rat MI models, elevating Treg cell numbers can prevent adverse ventricular morphological changes by reducing inflammation and directly protecting cardiomyocytes, thereby enhancing post-ischemic cardiac function (146, 147). Tregs may slow down the process of cardiac fibrosis by inhibiting the release of pro-fibrotic factors, which is crucial for the recovery of cardiac muscle function and structure (148, 149). Additionally, Treg cells support post-MI recovery by regulating the differentiation of monocytes and macrophages into reparative M2 macrophages, diminishing post-infarction inflammation, restraining excessive matrix degradation, and thus slowing adverse morphological changes (64, 150).Accumulating Treg cells in the injured murine heart participate in regulating fibroblast behavior and function, inhibiting post-MI fibrosis, and alleviating cardiac stiffening and dysfunction (151). One study showed that Ccl17 deficiency leads to reduced left ventricular remodeling post-MI and after angiotensin II and norepinephrine administration. This was associated with diminished myocardial fibrosis, cardiomyocyte hypertrophy, and improved left ventricular contractile function (152). The evidence demonstrated that Tregs mediated the protective effects of Ccl17 deficiency against myocardial inflammation and adverse left ventricular remodeling (153). Thus, inhibiting CCL17 might be an effective strategy to promote Treg recruitment and suppress myocardial inflammation.

Research has shown that MI patients have reduced Treg cell numbers, and lower circulating TREG cells correlate with increased MI risk (154).In murine MI models, externally administered Treg cells can reduce infarct size and ischemia-induced cardiac morphological changes. Furthermore, in mice undergoing coronary artery ligation and reperfusion, the selective depletion of Treg cells exacerbates ischemia-reperfusion injury (155, 156). Furthermore, in mice undergoing coronary artery ligation and reperfusion, the selective depletion of Treg cells exacerbates ischemia-reperfusion injury (157, 158). Treg cells might play a key protective role in myocardial ischemia, MI, and post-MI cardiac remodeling by protecting the heart, inhibiting excessive inflammatory responses, promoting repair, and improving function. Hence, modulating Treg cell numbers and function might offer novel strategies for the treatment of myocardial ischemia and infarction.

### 3.4 Rheumatic heart disease and valvular disorders

Rheumatic Heart Disease (RHD) is a sequelae of untreated rheumatic fever, predominantly affecting the heart valves (159, 160). RHD remains a significant cause of heart failure in developing countries, especially in areas lacking timely treatment for rheumatic fever (161, 162).

After a myocardial infarction, the heart needs to restore its blood supply, and Tregs may support this process by promoting angiogenesis. Treg cells play a pivotal role in immune modulation. A decline in the number of Treg cells in the peripheral blood of patients has been observed, and this decline is more pronounced when multiple valves are affected concurrently (163, 164).In RHD, the reduction in Treg cell count might signify a compromised immunoregulatory capacity, leading to persistent inflammatory responses and valvular damage, potentially exacerbating the progression of valvular diseases (165). The simultaneous impairment of multiple valves reflects heightened inflammatory reactions or disease severity. As the disease intensifies, the immunomodulatory function of Treg cells might further diminish.

By regulating inflammation and fibrosis, Tregs may help reduce the risk of recurrence of heart disease after myocardial infarction (166). While the proportion of Treg cells may vary in RHD, there isn’t a direct correlation with echocardiographically determined valvular thickness or hemodynamic alterations (164, 167). This suggests that structural and functional damages to the valves might not have a linear relationship with Treg cell quantity or proportion, implying potential interference from other factors. Treg cells possibly play a crucial role in the progression of RHD. In rheumatic heart disease, inflammation is a key factor leading to damage and dysfunction of the heart valves. Tregs can reduce inflammation of the heart valves through their anti-inflammatory action. Research and understanding of Treg cells may help us better understand the pathogenesis of rheumatic heart disease and provide new ideas and strategies for treatment (166). Tregs play an important role in the development of rheumatic heart disease and valvular diseases, especially through their immune-regulatory and anti-inflammatory functions. By controlling the inflammatory response and reducing tissue damage, Tregs help slow the progression of the disease (165). These findings offer a new perspective for the treatment of valvular diseases, especially in terms of immune-regulatory therapy.

### 3.5 Hypertension

Although hypertension has long been perceived as a “non-inflammatory” disease, mounting evidence highlights the central roles of the immune system and inflammation in its pathogenesis (168, 169). Chronic inflammation is considered an important factor in the development of hypertension, and Tregs may help reduce the risk of hypertension by suppressing chronic inflammatory responses (170). Chronic inflammation is considered an important factor in the development of hypertension, and Tregs may help reduce the risk of hypertension by suppressing chronic inflammatory responses (171). Activation and infiltration of T lymphocytes can be observed in numerous hypertension models, particularly within the kidneys and vessels (172). Studies suggest that T lymphocytes get activated and infiltrate target organs as blood pressure starts rising. Tregs can inhibit autoimmune reactions against vascular and renal tissues, reducing immune-mediated damage to these organs (173). The kidneys play a central role in regulating blood pressure, and Tregs may protect renal function by reducing renal inflammation and fibrosis, thereby combating hypertension (174). Cytokines and other inflammatory mediators released by these cells might promote vasoconstriction and cell proliferation, exacerbating hypertension (175). Transplantation of TREG cells can restore endothelial function.

Endothelial cells are fundamental regulators of vasoconstriction and vasodilation, and endothelial dysfunction is considered an early indicator and a trigger for hypertension, atherosclerosis, and vasculitis (176, 177).In experiments, after mice were administered aldosterone, their vascular endothelial function was compromised, evident as adverse vascular remodeling and elevated systolic blood pressure (SBP) (178). However, pre-injecting mice with TREG cells before aldosterone administration mitigated the SBP increase, thereby shielding vessels from damage (179).TREG cells might counteract vascular damage induced by angiotensin II; transplantation of TREG cells can rejuvenate endothelial function and slow the consistent rise of SBP (180). Tregs’ anti-inflammatory action may help maintain the health of the vascular endothelium and reduce endothelial dysfunction, which is crucial for the prevention of hypertension (181). The anti-inflammatory ability of Tregs can reduce inflammation of the blood vessel walls, helping to maintain normal vascular tone and blood pressure (182, 183).

Research indicates that Tregs can promote nitric oxide (NO) production, an essential endothelial relaxant crucial for appropriate vascular dilation (184). Tregs, by secreting anti-inflammatory cytokines like IL-10 and TGF-β, can inhibit the activity of other inflammatory cells, thereby conserving endothelial function (185). Additionally, studies report that TREG cells might release IL-10 to optimize microvascular endothelial function in hypertensive patients (173).IL-10 itself can reduce NADPH oxidase activity, thereby enhancing endothelial relaxation function (186). Upon transferring normal mice TREG cells into angiotensin II-treated IL-10 deficient mice, restoration of endothelial function and a decrease in SBP were observed (187).

Nevertheless, some studies present contradictory findings, suggesting that the immunosuppressive effects of transferred Treg cells ameliorate cardiac damage and improve electrical remodeling, yet the transplantation of TREG cells does not significantly influence blood pressure itself (188). The role of Tregs in hypertension and endothelial dysfunction remains under investigation, but they have been recognized as potential therapeutic targets.

### 3.6 Heart failure

The imbalance of the immune-inflammatory response plays a significant role in the progression of Chronic Heart Failure (CHF) (189). Immune activation and inflammation are involved in the progression of CHF, and an imbalance of Th17/Treg in CHF patients suggests that this imbalance plays a role in the pathogenesis (190). Balancing Th17/Treg may be a promising therapeutic approach for CHF patients (191). Studies have shown that catechins improve cardiac dysfunction in rats with chronic heart failure by regulating the balance between Th17 and Treg cells. Further results indicate that catechins can significantly inhibit immune activation and regulate the imbalance of IL-17/IL-10 levels (192). Therefore, catechins can reverse the abnormal polarization of TH17 and Treg in peripheral blood and spleen, improving the progression of chronic heart failure. Additionally, a key process in heart failure is cardiac fibrosis, and Treg cells slow down this process by regulating the expression of fibrosis-related cytokines (193). Anti-inflammatory cytokines produced by Treg cells, such as TGF-β and IL-10, can directly or indirectly inhibit the production of pro-fibrotic cytokines (194).

## 4 Potential therapeutic strategies targeting Treg cells in cardiovascular disease

In both *in vitro* and *in vivo* studies, the adoptive transfer or effective expansion of exogenous Treg cells has shown the potential to decelerate the progression of numerous cardiovascular diseases. As a result, this method is emerging as a potential therapeutic approach to cardiovascular diseases. Preliminary clinical data has reported on the efficacy and safety of targeting Treg cells through the expansion of human Treg cells ex *vivo*.

### 4.1 Immunomodulatory treatments:

The pro-inflammatory role of IL-1β in cardiovascular events and its interaction with the anti-inflammatory and immune-regulatory functions of Tregs constitute an important mechanism in the development of cardiovascular diseases (195). Tregs, by regulating the activity of IL-1β, can alleviate cardiac inflammation and promote healthier cardiac repair. These findings offer a new perspective for immune-regulatory treatment strategies in cardiovascular diseases (196). A randomized, double-blind trial investigated canakinumab (a therapeutic monoclonal antibody targeting IL-1β). The study, which encompassed 10,061 patients with a history of myocardial infarction, demonstrated that anti-inflammatory treatment targeting the IL-1β innate immune pathway using canakinumab significantly reduced the recurrence of cardiovascular events. When compared to the placebo group, the levels of high-sensitivity C-reactive protein in the 50mg canakinumab group showed a median reduction of 26 percentage points from baseline, 37 percentage points in the 150mg group, and 41 percentage points in the 300mg group1 (197). IL-1β may directly or indirectly affect the function and number of Tregs. For example, high levels of IL-1β may inhibit the activity of Tregs or promote the activation of inflammatory T cells, thereby impacting the inflammatory response (198). Consequently, immunomodulation targeting IL-1β offers significant protective effects against cardiovascular diseases.

IL-2 is a key factor for the survival and function of Tregs. IL-2 not only promotes the proliferation of Tregs but also maintains their suppressive function. IL-2 enhances the suppressive function of Tregs by activating specific signaling pathways, such as the STAT5 pathway (199). A randomized, double-blind, placebo-controlled phase I/II clinical trial treated patients with stable ischemic heart disease and acute coronary syndrome (LILACS) using low-dose IL-2. This trial employed Aldesleukin (a recombinant form of IL-2) to determine its safety, tolerability, and the dosage required to increase the average circulating Treg levels by at least 75% (200). Aldesleukin offers an intriguing alternative approach to achieving atheroprotective immunomodulation. The trial aimed to investigate the effects of low-dose IL treatment in augmenting Tregs. By supporting the function of Tregs, IL-2 helps reduce the inflammatory response in cardiovascular diseases, especially in conditions like atherosclerosis and myocardial infarction. Tregs, by alleviating inflammation, protect vascular endothelial function and reduce the formation of atherosclerotic plaques, thus contributing to cardiovascular health (201).

Experimental and epidemiological studies have backed the protective effects associated with various LDL-targeted antibodies (202). Apolipoprotein (apoB) is a specific lipoprotein, serving as the primary carrier of various lipids (like cholesterol) in the bloodstream (203). It can be found in two main lipoproteins: LDL and very low-density lipoprotein (VLDL). Elevated apoB levels are correlated with an increased risk of cardiovascular disease (97, 204). ApoB may affect the function of Tregs directly or indirectly. For instance, oxidized LDL may negatively impact Tregs, reducing their suppressive ability. ApoB, by affecting Tregs, can lead to an imbalance in immune regulation, potentially exacerbating cardiovascular inflammation and the progression of atherosclerosis (97, 205). Given that each LDL and VLDL particle contains just one apoB protein, the concentration of apoB in the blood can be regarded as a representative of lipoprotein particles with potential atherogenic properties (206, 207). Building on these promising observations, Lehrer et al. tested a monoclonal antibody targeting oxidized LDL (oxLDL), MLDL1278A, for its potential to reduce inflammation in atherosclerosis (208).In the study, 147 atherosclerotic patients with inflammation in the carotid or aorta plaques were randomly divided into three groups. Results indicated that while the MLDL1278A-treated groups had higher serum concentrations, there wasn’t a significant reduction in arteritis compared to the placebo group. MLDL1278A was well-tolerated, with no immunogenicity observed. In multiple dosing groups, levels of tumor necrosis factor-α and interleukin-6 showed a slight increase by the fourth week.

CTLA-4 (Cytotoxic T-lymphocyte-associated protein 4) is a crucial molecule on the surface of Treg cells. By binding to the B7 family molecules on antigen-presenting cells, CTLA-4 dependent downregulation can inhibit T-cell activation. CTLA-4 blockade triggers an over-proliferation of CD28-dependent Treg cells, and a concurrent inactivation of Treg cells is necessary for tumor rejection responses (209). Therefore, Treg cells self-regulate through a CTLA-4 and CD28-dependent feedback loop. The disruption of this loop by CTLA-4 blockade could counteract the damage caused by an overly activated immune-inflammatory response to cardiac and vascular tissues, making it a potential target for the treatment of certain types of cardiovascular diseases. Ipilimumab, an antibody drug targeting CTLA-4, is commonly used for certain types of cancer treatment, but its role in modulating Treg cells also shows potential. Treatment with Ipilimumab leads to a reduction in Treg cells mediated by macrophage ADCC, while also shifting TAM polarization from M2 to M1, subsequently attracting CD8 cells and increasing the anti-tumor response (210). GITR (Glucocorticoid-Induced TNFR-Related protein) also plays a role in the regulation of Treg cells and can influence immune responses. Some studies are exploring therapies targeting to modulate Treg cell functions (211).

### 4.2 Active immunotherapy using vaccines

The therapeutic use of PCSK9 antibodies has been shown to effectively lower LDL cholesterol levels, and when combined with statins, it has been proven to further reduce the risk of cardiovascular diseases (212). However, the high cost of this treatment has limited its widespread use among patients. Therefore, the potential to induce similar antibodies through vaccination is being explored as a more cost-effective approach.

An atherosclerosis vaccine based on the principle of modulating the autoimmune response against LDL-related antigens has been tested in clinical trials. This vaccine may activate or increase the number of Tregs, thereby enhancing their regulatory role in cardiovascular inflammation (99). By modulating the activity of Tregs, the vaccine could alter the immune response to LDL or oxidized LDL (oxLDL), reducing the development of atherosclerosis (213). According to experimental evidence, the primary mode of action of such vaccines is to inhibit the Th1-dependent pro-inflammatory immune response against antigens formed in modified LDL particles (110). This inhibition is mediated by antigen-specific regulatory T cells, which are activated when macrophages and other antigen-presenting cells in atherosclerotic plaques are exposed to their homologous antigens (214). Theoretically, these antigen-specific Tregs not only suppress the activity of Th1 T cells with corresponding antigen specificity but also inhibit plaque inflammation by releasing anti-inflammatory cytokines, such as IL-10 and TGF-β. The advantage of this mode of action is a lower risk of adverse side effects associated with systemic anti-inflammatory treatments. Therefore, it is expected not to lead to the slight increase in fatal infection frequency observed in the CANTOS trial (215). It has been proven in experimental models that atherosclerosis can be suppressed by activating Tregs. In the long term, a vaccine specifically inducing LDL tolerance in atherosclerotic plaques is still considered the best alternative treatment option.

A novel anti-PCSK9 vaccine formulation, termed L-IFPTA, was developed to induce the host to produce anti-PCSK9 antibodies, thereby lowering LDL-C levels in mice. This vaccine induces functional anti-PCSK9 antibodies, effectively blocking the interaction between PCSK9 and LDLR, increasing the expression of LDLR on hepatocyte surfaces, and enhancing cholesterol clearance from the bloodstream (216).The L-FPTA vaccine can also modulate the balance of the immune system, reducing levels of the pro-inflammatory factor IFN-c, and increasing levels of anti-inflammatory factors L-4 and L-10. The L-FPTA vaccine may indirectly enhance the function of Tregs by raising levels of L-10, thereby more effectively suppressing inflammation related to cardiovascular diseases. Adjusting the immune environment may impact the number and activity of Tregs, thereby altering the progression of cardiovascular diseases. The L-IFPTA vaccine significantly reduced TC, LDL-C, and VLDL-C levels in mice, diminishing the formation and severity of atherosclerotic plaques. In a preclinical study on healthy non-human primates to determine the immunogenicity and safety of the “liposomal immunogenic fusion PCSK9-tetanus toxoid adjuvant” (L-IFPTA) nanoliposome anti-PCSK9 vaccine, the data suggested that the L-IFPTA vaccine potently and safely induced these primates to produce functional anti-PCSK9 antibodies (217). This vaccine effectively stimulates a humoral immune response, generating inhibitory antibodies against plasma PCSK9, without causing systemic inflammation or adverse effects on organ functions. It also modulates the immune system balance by decreasing pro-inflammatory IFN-c levels and increasing anti-inflammatory IL-4 and IL-10 levels. The L-IFPTA vaccine demonstrates good safety and tolerability (218).

Currently, the safety of PCSK9 peptide vaccines, the ability of PCSK9 antibodies to respond and reduce LDL, has been tested in phase I trials. Randomized placebo-controlled clinical trials evaluating anti-inflammatory drugs elucidate whether targeting inflammation itself would reduce cardiovascular events and risks. AT04A and AT06A are two AFFITOPE^®^ peptide candidate vaccines under development, aiming to treat hypercholesterolemia by inducing specific antibodies against proprotein convertase subtilisin/kexin type 9. This phase I, single-blind, randomized, placebo-controlled study was conducted among 72 healthy participants with baseline fasting LDLc levels averaging 117.1 mg/d. Throughout the study period, the AT04A group exhibited an average reduction of -7.2% in LDL levels compared to the placebo group (219). Such antibodies help clear pathogenic particles from circulation. They can also neutralize pathogenic particles in the extracellular space, preventing their binding to pattern recognition receptors, reducing intracellular cholesterol accumulation, and inhibiting the induction of pro-inflammatory signals in macrophages. High levels of cholesterol within cells can impair the function and stability of Tregs. By reducing intracellular cholesterol accumulation, Tregs can maintain their functionality and ability to regulate the immune system effectively (99). Cholesterol metabolism is crucial for cell membrane integrity and signaling. By regulating cholesterol levels within Tregs, it’s possible to influence their survival, proliferation, and capacity to suppress pro-inflammatory responses (220). Reducing cholesterol accumulation in Tregs can improve their ability to control the inflammatory processes that contribute to plaque development and stability (221).

### 4.3 Immune adsorption therapy

Transgenic mice expressing the Tumor Necrosis Factor-α (TNF-α) gene under the cardiomyocyte promoter (TNF1.6 mice) develop dilated cardiomyopathy (DCM). These transgenic mice exhibit widespread cardiac inflammation, suggesting that an immunopathogenic mechanism might promote cardiomyopathy. Compared to control TNF1.6 mice, TNF1.6 mice treated with monoclonal anti-CD3 or anti-CD4 antibodies displayed significant reductions in heart size and plasma troponin I concentrations due to T cell depletion. Adoptive transfer of CD4(+)CD25(+) cells from H310A1-infected mice into uninfected TNF1.6 recipients eliminated cardiomyopathy. Administration of recombinant TNF-α exogenously to H310A1-infected mice for 4 days abrogated immune suppression (222). After six months of immune adsorption therapy, the left ventricular ejection fraction in DCM patients significantly improved, correlating closely with an increase in peripheral Treg cell numbers. Experimental evidence suggests that direct transfer of Treg cells into TNF transgenic mice can reduce their heart weight and plasma levels of troponin I, alleviating DCM symptoms. Compared to healthy individuals, the number of Treg cells in the myocardium of DCM patients is notably reduced (140).In this study, induced glucocorticoid tumor necrosis factor R-related protein was used as a marker for Treg cells rather than the more specific FOXP3 transcription factor. In summary, autoimmunity modulation might be beneficial in preventing myocarditis and subsequent DCM, and manipulating the number and function of Treg cells could be a promising strategy against these challenging diseases.

## 5 Conclusion and prospective

While certain cardiovascular diseases such as atherosclerosis, hypertension, and myocardial infarction are not considered classical autoimmune diseases, immune responses related to self-antigens indeed play significant roles in their development (223). Specifically, T cells, especially Treg cells, occupy a central position in the pathophysiology of these diseases (224). Studies indicate that defects in the number and function of Treg cells are associated with a variety of cardiovascular diseases, and enhancing the function or number of Treg cells can effectively slow down disease progression (225). Hence, Treg cells are viewed as potential therapeutic targets. Ample experimental and clinical research suggests that a reduced number and impaired function of Treg cells might be present in multiple cardiovascular diseases (15). Adoptive transfer of exogenous Treg cells or expansion of endogenous Treg cells effectively inhibited the progression of many cardiovascular diseases (97). Although the therapeutic mechanisms of Treg cells for cardiovascular diseases remain not fully elucidated, they provide a promising research direction, potentially revealing immune-regulatory mechanisms in cardiovascular diseases.

Experimental studies have granted us many new insights into cardiovascular diseases. However, transitioning from these experimental findings to actual clinical applications often requires considerable time, given that clinical studies must consider numerous variables and complexities. In an inflammatory environment, Tregs may lose their suppressive function or transform into other types of T cells. Tregs must precisely target inflammation related to cardiovascular diseases, avoiding widespread suppression of the immune system, which could lead to infections and other adverse consequences (226). Effectively expanding and maintaining the function of Tregs *in vitro* and efficiently delivering Tregs to specific areas of cardiovascular disease present additional technical challenges. Most experimental research on cardiovascular disease vaccines focuses on early prevention. Yet, for patients in advanced stages exhibiting clinical symptoms, these study results might not be applicable. Developing methods for selectively activating or enhancing the function of Tregs, especially at sites of inflammation related to cardiovascular diseases, is important (227). Utilizing CRISPR or other gene-editing tools to enhance the stability and specificity of Tregs is another approach. Improving *in vitro* culture conditions to increase the quantity and quality of Tregs, including the use of specific cytokines and culture media, is also crucial (228). Developing new delivery systems, such as biocompatible materials and nanoparticles, can improve the stability and targeting of Tregs within the body. Combining Treg therapy with other treatment methods, such as lipid-lowering drugs or anti-inflammatory therapies, can enhance the effectiveness of the treatment (229). To translate into stable treatment regimens for cardiovascular diseases, comprehensive clinical trials are needed to understand the vaccine’s mechanism of action, monitor vaccine responses, evaluate efficacy in advanced cardiovascular diseases, and assess potential safety concerns.

## Author contributions

YX: Writing – original draft, Writing – review & editing. DG: Writing – review & editing. XW: Writing – review & editing. BL: Writing – review & editing. XS: Writing – review & editing, Validation. YS: Funding acquisition, Writing – review & editing. DM: Writing – review & editing, Supervision, Conceptualization, Validation, Visualization.

## References

[1] Xia S Du X Guo L Du J Arnott C Lam CSP. Sex differences in primary and secondary prevention of cardiovascular disease in China. Circulation. (2020) 141:530–9. doi: 10.1161/CIRCULATIONAHA.119.043731 32065775

[2] Muntner P Whittle J Lynch AI Colantonio LD Simpson LM Einhorn PT. Visit-to-visit variability of blood pressure and coronary heart disease, stroke, heart failure, and mortality: A cohort study. Ann Intern Med. (2015) 163:329–38. doi: 10.7326/M14-2803 PMC5021508 26215765

[3] Axelrod ML Meijers WC Screever EM Qin J Carroll MG Sun X. T cells specific for α-myosin drive immunotherapy-related myocarditis. Nature. (2022) 611:818–26. doi: 10.1038/s41586-022-05432-3 PMC9930174 36385524

[4] Fernandez DM Rahman AH Fernandez NF Chudnovskiy A Amir ED Amadori L. Single-cell immune landscape of human atherosclerotic plaques. Nat Med. (2019) 25:1576–88. doi: 10.1038/s41591-019-0590-4 PMC7318784 31591603

[5] Rurik JG Tombácz I Yadegari A Méndez Fernández PO Shewale SV Li L. CAR T cells produced in *vivo* to treat cardiac injury. Science. (2022) 375:91–6. doi: 10.1126/science.abm0594 PMC9983611 34990237

[6] Joller N Lozano E Burkett PR Patel B Xiao S Zhu C. Treg cells expressing the coinhibitory molecule TIGIT selectively inhibit proinflammatory Th1 and Th17 cell responses. Immunity. (2014) 40:569–81. doi: 10.1016/j.immuni.2014.02.012 PMC4070748 24745333

[7] Dikiy S Rudensky AY. Principles of regulatory T cell function. Immunity. (2023) 56:240–55. doi: 10.1016/j.immuni.2023.01.004 36792571

[8] Singer BD D’Alessio FR. Comment on Adamzik et al.: An increased alveolar CD4 + CD25 + Foxp3 + T-regulatory cell ratio in acute respiratory distress syndrome is associated with increased 30-day mortality. Intensive Care Med. (2014) 40:1604. doi: 10.1007/s00134-014-3399-0 25030097 PMC4177340

[9] Peters J. An increased alveolar CD4+ CD25+ Foxp3+ T-regulatory cell ratio in acute respiratory distress syndrome is associated with increased 30-day mortality: response to comment by Singer and D’Alessio. Intensive Care Med. (2014) 40:1605–6. doi: 10.1007/s00134-014-3437-y 25164394

[10] van der Veeken J Campbell C Pritykin Y Schizas M Verter J Hu W. Genetic tracing reveals transcription factor Foxp3-dependent and Foxp3-independent functionality of peripherally induced Treg cells. Immunity. (2022) 55:1173–1184.e7. doi: 10.1016/j.immuni.2022.05.010 35700740 PMC9885886

[11] Ohkura N Sakaguchi S. Transcriptional and epigenetic basis of Treg cell development and function: its genetic anomalies or variations in autoimmune diseases. Cell Res. (2020) 30:465–74. doi: 10.1038/s41422-020-0324-7 PMC7264322 32367041

[12] Wing JB Tanaka A Sakaguchi S. Human FOXP3(+) regulatory T cell heterogeneity and function in autoimmunity and cancer. Immunity. (2019) 50:302–16. doi: 10.1016/j.immuni.2019.01.020 30784578

[13] Lu L Barbi J Pan F. The regulation of immune tolerance by FOXP3. Nat Rev Immunol. (2017) 17:703–17. doi: 10.1038/nri.2017.75 PMC5793224 28757603

[14] Cortez JT Montauti E Shifrut E Gatchalian J Zhang Y Shaked O. CRISPR screen in regulatory T cells reveals modulators of Foxp3. Nature. (2020) 582:416–20. doi: 10.1038/s41586-020-2246-4 PMC7305989 32499641

[15] Xia N Lu Y Gu M Li N Liu M Jiao J. A unique population of regulatory T cells in heart potentiates cardiac protection from myocardial infarction. Circulation. (2020) 142:1956–73. doi: 10.1161/CIRCULATIONAHA.120.046789 32985264

[16] Bansal SS Ismahil MA Goel M Zhou G Rokosh G Hamid T. Dysfunctional and proinflammatory regulatory T-lymphocytes are essential for adverse cardiac remodeling in ischemic cardiomyopathy. Circulation. (2019) 139:206–21. doi: 10.1161/CIRCULATIONAHA.118.036065 PMC6322956 30586716

[17] Turillazzi E Di Paolo M Neri M Riezzo I Fineschi V. A theoretical timeline for myocardial infarction: immunohistochemical evaluation and western blot quantification for Interleukin-15 and Monocyte chemotactic protein-1 as very early markers. J Transl Med. (2014) 12:188. doi: 10.1186/1479-5876-12-188 24989171 PMC4094437

[18] Zhao K Tang M Wang H Zhou Z Wu Y Liu S. Simultaneous detection of three biomarkers related to acute myocardial infarction based on immunosensing biochip. Biosens Bioelectron. (2019) 126:767–72. doi: 10.1016/j.bios.2018.11.044 30554098

[19] Tang J Cui X Caranasos TG Hensley MT Vandergriff AC Hartanto Y. Heart repair using nanogel-encapsulated human cardiac stem cells in mice and pigs with myocardial infarction. ACS Nano. (2017) 11:9738–49. doi: 10.1021/acsnano.7b01008 PMC5656981 28929735

[20] Ciuffreda MC Malpasso G Chokoza C Bezuidenhout D Goetsch KP Mura M. Synthetic extracellular matrix mimic hydrogel improves efficacy of mesenchymal stromal cell therapy for ischemic cardiomyopathy. Acta Biomater. (2018) 70:71–83. doi: 10.1016/j.actbio.2018.01.005 29341932

[21] Tanaka A Sakaguchi S. Targeting Treg cells in cancer immunotherapy. Eur J Immunol. (2019) 49:1140–6. doi: 10.1002/eji.201847659 31257581

[22] Tanaka A Sakaguchi S. Regulatory T cells in cancer immunotherapy. Cell Res. (2017) 27:109–18. doi: 10.1038/cr.2016.151 PMC5223231 27995907

[23] Ference BA Ginsberg HN Graham I Ray KK Packard CJ Bruckert E. Low-density lipoproteins cause atherosclerotic cardiovascular disease. 1. Evidence from genetic, epidemiologic, and clinical studies. A consensus statement from the European Atherosclerosis Society Consensus Panel. Eur Heart J. (2017) 38:2459–72. doi: 10.1093/eurheartj/ehx144 PMC5837225 28444290

[24] Navarese EP Robinson JG Kowalewski M Kolodziejczak M Andreotti F Bliden K. Association between baseline LDL-C level and total and cardiovascular mortality after LDL-C lowering: A systematic review and meta-analysis. Jama. (2018) 319:1566–79. doi: 10.1001/jama.2018.2525 PMC5933331 29677301

[25] Ray KK Landmesser U Leiter LA Kallend D Dufour R Karakas M. Inclisiran in patients at high cardiovascular risk with elevated LDL cholesterol. N Engl J Med. (2017) 376:1430–40. doi: 10.1056/NEJMoa1615758 28306389

[26] Huang L Chambliss KL Gao X Yuhanna IS Behling-Kelly E Bergaya S. SR-B1 drives endothelial cell LDL transcytosis via DOCK4 to promote atherosclerosis. Nature. (2019) 569:565–9. doi: 10.1038/s41586-019-1140-4 PMC6631346 31019307

[27] Esterházy D Loschko J London M Jove V Oliveira TY Mucida D. Classical dendritic cells are required for dietary antigen-mediated induction of peripheral T(reg) cells and tolerance. Nat Immunol. (2016) 17:545–55. doi: 10.1038/ni.3408 PMC4837106 27019226

[28] Perry JSA Lio CJ Kau AL Nutsch K Yang Z Gordon JI. Distinct contributions of Aire and antigen-presenting-cell subsets to the generation of self-tolerance in the thymus. Immunity. (2014) 41:414–26. doi: 10.1016/j.immuni.2014.08.007 PMC4175925 25220213

[29] Weist BM Kurd N Boussier J Chan SW Robey EA. Thymic regulatory T cell niche size is dictated by limiting IL-2 from antigen-bearing dendritic cells and feedback competition. Nat Immunol. (2015) 16:635–41. doi: 10.1038/ni.3171 PMC4439282 25939026

[30] Kong KF Fu G Zhang Y Yokosuka T Casas J Canonigo-Balancio AJ. Protein kinase C-η controls CTLA-4-mediated regulatory T cell function. Nat Immunol. (2014) 15:465–72. doi: 10.1038/ni.2866 PMC4040250 24705298

[31] Proto JD Doran AC Gusarova G Yurdagul A Jr. Sozen E Subramanian M. Regulatory T cells promote macrophage efferocytosis during inflammation resolution. Immunity. (2018) 49:666–677.e6. doi: 10.1016/j.immuni.2018.07.015 30291029 PMC6192849

[32] Sawant DV Yano H Chikina M Zhang Q Liao M Liu C. Adaptive plasticity of IL-10(+) and IL-35(+) T(reg) cells cooperatively promotes tumor T cell exhaustion. Nat Immunol. (2019) 20:724–35. doi: 10.1038/s41590-019-0346-9 PMC6531353 30936494

[33] Kanamori M Nakatsukasa H Okada M Lu Q Yoshimura A. Induced regulatory T cells: their development, stability, and applications. Trends Immunol. (2016) 37:803–11. doi: 10.1016/j.it.2016.08.012 27623114

[34] Turner JA Stephen-Victor E Wang S Rivas MN Abdel-Gadir A Harb H. Regulatory T cell-derived TGF-β1 controls multiple checkpoints governing allergy and autoimmunity. Immunity. (2020) 53:1202–1214.e6. doi: 10.1016/j.immuni.2020.10.002 33086036 PMC7744401

[35] Montauti E Weinberg SE Chu P Chaudhuri S Mani NL Iyer R. A deubiquitination module essential for T(reg) fitness in the tumor microenvironment. Sci Adv. (2022) 8:eabo4116. doi: 10.1126/sciadv.abo4116 36427305 PMC9699683

[36] Lainé A Labiad O Hernandez-Vargas H This S Sanlaville A Léon S. Regulatory T cells promote cancer immune-escape through integrin αvβ8-mediated TGF-β activation. Nat Commun. (2021) 12:6228. doi: 10.1038/s41467-021-26352-2 34711823 PMC8553942

[37] Field CS Baixauli F Kyle RL Puleston DJ Cameron AM Sanin DE. Mitochondrial integrity regulated by lipid metabolism is a cell-intrinsic checkpoint for Treg suppressive function. Cell Metab. (2020) 31:422–437.e5. doi: 10.1016/j.cmet.2019.11.021 31883840 PMC7001036

[38] Zhang ZT Zhang DY Xie K Wang CJ Xu F. Luteolin activates Tregs to promote IL-10 expression and alleviating caspase-11-dependent pyroptosis in sepsis-induced lung injury. Int Immunopharmacol. (2021) 99:107914. doi: 10.1016/j.intimp.2021.107914 34246059

[39] Kuan R Agrawal DK Thankam FG. Treg cells in atherosclerosis. Mol Biol Rep. (2021) 48:4897–910. doi: 10.1007/s11033-021-06483-x 34117978

[40] Rubtsov YP Rasmussen JP Chi EY Fontenot J Castelli L Ye X. Regulatory T cell-derived interleukin-10 limits inflammation at environmental interfaces. Immunity. (2008) 28:546–58. doi: 10.1016/j.immuni.2008.02.017 18387831

[41] Shao Y Yang WY Saaoud F C.t. Drummer Y Xu K Lu Y. IL-35 promotes CD4+Foxp3+ Tregs and inhibits atherosclerosis via maintaining CCR5-amplified Treg-suppressive mechanisms. JCI Insight. (2021) 6(19):e152511. doi: 10.1172/jci.insight.152511 34622804 PMC8525592

[42] Sullivan JA Tomita Y Jankowska-Gan E Lema DA Arvedson MP Nair A. Treg-cell-derived IL-35-coated extracellular vesicles promote infectious tolerance. Cell Rep. (2020) 30:1039–1051.e5. doi: 10.1016/j.celrep.2019.12.081 31995748 PMC7042971

[43] Hernandez R Põder J LaPorte KM Malek TR. Engineering IL-2 for immunotherapy of autoimmunity and cancer. Nat Rev Immunol. (2022) 22:614–28. doi: 10.1038/s41577-022-00680-w 35217787

[44] Abbas AK Trotta E. Revisiting IL-2: Biology and therapeutic prospects. Sci Immunol. (2018) 3(25):eaat1482. doi: 10.1126/sciimmunol.aat1482 29980618

[45] Spolski R Li P Leonard WJ. Biology and regulation of IL-2: from molecular mechanisms to human therapy. Nat Rev Immunol. (2018) 18:648–59. doi: 10.1038/s41577-018-0046-y 30089912

[46] Arenas-Ramirez N Woytschak J Boyman O. Interleukin-2: biology, design and application. Trends Immunol. (2015) 36:763–77. doi: 10.1016/j.it.2015.10.003 26572555

[47] Apert C Romagnoli P van Meerwijk JPM. IL-2 and IL-15 dependent thymic development of Foxp3-expressing regulatory T lymphocytes. Protein Cell. (2018) 9:322–32. doi: 10.1007/s13238-017-0425-3 PMC5876181 28540653

[48] Sharabi A Li H Kasper IR Pan W Meidan E Tsokos MG. PP2A enables IL-2 signaling by preserving IL-2Rβ chain expression during Treg development. JCI Insight. (2019) 5(9):e126294. doi: 10.1172/jci.insight.126294 30912768 PMC6538314

[49] Apert C Galindo-Albarrán AO Castan S Detraves C Michaud H McJannett N. IL-2 and IL-15 drive intrathymic development of distinct periphery-seeding CD4(+)Foxp3(+) regulatory T lymphocytes. Front Immunol. (2022) 13:965303. doi: 10.3389/fimmu.2022.965303 36159793 PMC9495261

[50] Wong HS Park K Gola A Baptista AP Miller CH Deep D. A local regulatory T cell feedback circuit maintains immune homeostasis by pruning self-activated T cells. Cell. (2021) 184:3981–3997.e22. doi: 10.1016/j.cell.2021.05.028 34157301 PMC8390950

[51] Durand M Dubois F Dejou C Durand E Danger R Chesneau M. Increased degradation of ATP is driven by memory regulatory T cells in kidney transplantation tolerance. Kidney Int. (2018) 93:1154–64. doi: 10.1016/j.kint.2017.12.004 29455908

[52] Maciolek JA Pasternak JA Wilson HL. Metabolism of activated T lymphocytes. Curr Opin Immunol. (2014) 27:60–74. doi: 10.1016/j.coi.2014.01.006 24556090

[53] Zhang X Wang Y Qu GA,C Chen J. Pan-cancer analysis of PARP1 alterations as biomarkers in the prediction of immunotherapeutic effects and the association of its expression levels and immunotherapy signatures. Front Immunol. (2021) 12:721030. doi: 10.3389/fimmu.2021.721030 34531868 PMC8438309

[54] Bencsics M Bányai B Ke H Csépányi-Kömi R Sasvári P Dantzer F. PARP2 downregulation in T cells ameliorates lipopolysaccharide-induced inflammation of the large intestine. Front Immunol. (2023) 14:1135410. doi: 10.3389/fimmu.2023.1135410 37457706 PMC10347374

[55] Noh MY Lee WM Lee SJ Kim HY Kim SH Kim YS. Regulatory T cells increase after treatment with poly (ADP-ribose) polymerase-1 inhibitor in ischemic stroke patients. Int Immunopharmacol. (2018) 60:104–10. doi: 10.1016/j.intimp.2018.04.043 29709770

[56] Bastid J Regairaz A Bonnefoy N Déjou C Giustiniani J Laheurte C. Inhibition of CD39 enzymatic function at the surface of tumor cells alleviates their immunosuppressive activity. Cancer Immunol Res. (2015) 3:254–65. doi: 10.1158/2326-6066.CIR-14-0018 25403716

[57] De Marchi E Pegoraro A Turiello R Di Virgilio F Morello S Adinolfi E. A2A receptor contributes to tumor progression in P2X7 null mice. Front Cell Dev Biol. (2022) 10:876510. doi: 10.3389/fcell.2022.876510 35663396 PMC9159855

[58] Wang L Wan H Tang W Ni Y Hou X Pan L. Critical roles of adenosine A2A receptor in regulating the balance of Treg/Th17 cells in allergic asthma. Clin Respir J. (2018) 12:149–57. doi: 10.1111/crj.12503 27216911

[59] Liu Y Zheng P. Preserving the CTLA-4 checkpoint for safer and more effective cancer immunotherapy. Trends Pharmacol Sci. (2020) 41:4–12. doi: 10.1016/j.tips.2019.11.003 31836191 PMC7210725

[60] Dorta-Estremera S Hegde VL Slay RB Sun R Yanamandra AV Nicholas C. Targeting interferon signaling and CTLA-4 enhance the therapeutic efficacy of anti-PD-1 immunotherapy in preclinical model of HPV(+) oral cancer. J Immunother Cancer. (2019) 7:252. doi: 10.1186/s40425-019-0728-4 31533840 PMC6749627

[61] Nam JH Lee JH Choi SY Jung NC Song JY Seo HG. Functional ambivalence of dendritic cells: tolerogenicity and immunogenicity. Int J Mol Sci. (2021) 22(9):4430. doi: 10.3390/ijms22094430 33922658 PMC8122871

[62] Chang CY You R Armstrong D Bandi A Cheng YT Burkhardt PM. Chronic exposure to carbon black ultrafine particles reprograms macrophage metabolism and accelerates lung cancer. Sci Adv. (2022) 8:eabq0615. doi: 10.1126/sciadv.abq0615 36383649 PMC9668323

[63] Tekguc M Wing JB Osaki M Long J Sakaguchi S. Treg-expressed CTLA-4 depletes CD80/CD86 by trogocytosis, releasing free PD-L1 on antigen-presenting cells. Proc Natl Acad Sci U.S.A. (2021) 118(30):e2023739118. doi: 10.1073/pnas.2023739118 34301886 PMC8325248

[64] Zhu D Liu S Huang K Wang Z Hu S Li J. Intrapericardial exosome therapy dampens cardiac injury via activating Foxo3. Circ Res. (2022) 131:e135–50. doi: 10.1161/CIRCRESAHA.122.321384 PMC9667926 36252111

[65] Trzupek D Dunstan M Cutler AJ Lee M Godfrey L Jarvis L. Discovery of CD80 and CD86 as recent activation markers on regulatory T cells by protein-RNA single-cell analysis. Genome Med. (2020) 12:55. doi: 10.1186/s13073-020-00756-z 32580776 PMC7315544

[66] Soskic B Jeffery LE Kennedy A Gardner DH Hou TZ Halliday N. CD80 on human T cells is associated with FoxP3 expression and supports Treg homeostasis. Front Immunol. (2020) 11:577655. doi: 10.3389/fimmu.2020.577655 33488578 PMC7820758

[67] Ellis SD McGovern JL van Maurik A Howe D Ehrenstein MR Notley CA. Induced CD8+FoxP3+ Treg cells in rheumatoid arthritis are modulated by p38 phosphorylation and monocytes expressing membrane tumor necrosis factor α and CD86. Arthritis Rheumatol. (2014) 66:2694–705. doi: 10.1002/art.38761 24980778

[68] Sakaguchi S Mikami N Wing JB Tanaka A Ichiyama K Ohkura N. Regulatory T cells and human disease. Annu Rev Immunol. (2020) 38:541–66. doi: 10.1146/annurev-immunol-042718-041717 32017635

[69] Tay C Tanaka A Sakaguchi S. Tumor-infiltrating regulatory T cells as targets of cancer immunotherapy. Cancer Cell. (2023) 41:450–65. doi: 10.1016/j.ccell.2023.02.014 36917950

[70] Bae J Accardi F Hideshima T Tai YT Prabhala R Shambley A. Targeting LAG3/GAL-3 to overcome immunosuppression and enhance anti-tumor immune responses in multiple myeloma. Leukemia. (2022) 36:138–54. doi: 10.1038/s41375-021-01301-6 PMC8727303 34290359

[71] Bauché D Joyce-Shaikh B Jain R Grein J Ku KS Blumenschein WM. LAG3(+) regulatory T cells restrain interleukin-23-producing CX3CR1(+) gut-resident macrophages during group 3 innate lymphoid cell-driven colitis. Immunity. (2018) 49:342–352.e5. doi: 10.1016/j.immuni.2018.07.007 30097293

[72] Mulholland M Kritikou E Katra P Nilsson J Björkbacka H Lichtman AH. LAG3 regulates T cell activation and plaque infiltration in atherosclerotic mice. JACC Cardio Oncol. (2022) 4:635–45. doi: 10.1016/j.jaccao.2022.09.005 PMC9830219 36636446

[73] Dykema AG Zhang J Cheung LS Connor S Zhang B Zeng Z. Lung tumor-infiltrating T(reg) have divergent transcriptional profiles and function linked to checkpoint blockade response. Sci Immunol. (2023) 8:eadg1487. doi: 10.1126/sciimmunol.adg1487 37713507 PMC10629528

[74] Eskandari SK Allos H Al Dulaijan BS Melhem G Sulkaj I Alhaddad JB. mTORC1 inhibition protects human regulatory T cells from granzyme-B-induced apoptosis. Front Immunol. (2022) 13:899975. doi: 10.3389/fimmu.2022.899975 35757726 PMC9229986

[75] Christiansen D Mouhtouris E Hodgson R Sutton VR Trapani JA Ierino FL. Antigen-specific CD4(+) CD25(+) T cells induced by locally expressed ICOS-Ig: the role of Foxp3, Perforin, Granzyme B and IL-10 - an experimental study. Transpl Int. (2019) 32:1203–15. doi: 10.1111/tri.13474 31225919

[76] Wei J Wu D Shao Y Guo B Jiang J Chen J. ApoE-mediated systemic nanodelivery of granzyme B and CpG for enhanced glioma immunotherapy. J Control Release. (2022) 347:68–77. doi: 10.1016/j.jconrel.2022.04.048 35513207

[77] Sun B Liu M Cui M Li T. Granzyme B-expressing treg cells are enriched in colorectal cancer and present the potential to eliminate autologous T conventional cells. Immunol Lett. (2020) 217:7–14. doi: 10.1016/j.imlet.2019.10.007 31669380

[78] Wang RX Yu CR Dambuza IM Mahdi RM Dolinska MB Sergeev YV. Interleukin-35 induces regulatory B cells that suppress autoimmune disease. Nat Med. (2014) 20:633–41. doi: 10.1038/nm.3554 PMC4048323 24743305

[79] Sage PT Paterson AM Lovitch SB Sharpe AH. The coinhibitory receptor CTLA-4 controls B cell responses by modulating T follicular helper, T follicular regulatory, and T regulatory cells. Immunity. (2014) 41:1026–39. doi: 10.1016/j.immuni.2014.12.005 PMC4309019 25526313

[80] Wing JB Ise W Kurosaki T Sakaguchi S. Regulatory T cells control antigen-specific expansion of Tfh cell number and humoral immune responses via the coreceptor CTLA-4. Immunity. (2014) 41:1013–25. doi: 10.1016/j.immuni.2014.12.006 25526312

[81] Hu D Mohanta SK Yin C Peng L Ma Z Srikakulapu P. Artery Tertiary Lymphoid Organs Control Aorta Immunity and Protect against Atherosclerosis via Vascular Smooth Muscle Cell Lymphotoxin β Receptors. Immunity. (2015) 42:1100–15. doi: 10.1016/j.immuni.2015.05.015 PMC4678289 26084025

[82] Hasan MM Nair SS O’Leary JG Thompson-Snipes L Nyarige V Wang J. Implication of TIGIT(+) human memory B cells in immune regulation. Nat Commun. (2021) 12:1534. doi: 10.1038/s41467-021-21413-y 33750787 PMC7943800

[83] Bolivar-Wagers S Loschi ML Jin S Thangavelu G Larson JH McDonald-Hyman CS. Murine CAR19 Tregs suppress acute graft-versus-host disease and maintain graft-versus-tumor responses. JCI Insight. (2022) 7(17):e160674. doi: 10.1172/jci.insight.160674 35917188 PMC9536261

[84] Court AC Le-Gatt A Luz-Crawford P Parra E Aliaga-Tobar V Bátiz LF. Mitochondrial transfer from MSCs to T cells induces Treg differentiation and restricts inflammatory response. EMBO Rep. (2020) 21:e48052. doi: 10.15252/embr.201948052 31984629 PMC7001501

[85] Yamada Y Sugita S Horie S Yamagami S Mochizuki M. Mechanisms of immune suppression for CD8+ T cells by human corneal endothelial cells via membrane-bound TGFbeta. Invest Ophthalmol Vis Sci. (2010) 51:2548–57. doi: 10.1167/iovs.09-4233 20007827

[86] Motz GT Santoro SP Wang LP Garrabrant T Lastra RR Hagemann IS. Tumor endothelium FasL establishes a selective immune barrier promoting tolerance in tumors. Nat Med. (2014) 20:607–15. doi: 10.1038/nm.3541 PMC4060245 24793239

[87] Oh S Shim M Son M Jang JT Son KH Byun K. Attenuating effects of dieckol on endothelial cell dysfunction via modulation of Th17/Treg balance in the intestine and aorta of spontaneously hypertensive rats. Antioxid (Basel). (2021) 10(2):298. doi: 10.3390/antiox10020298 PMC7920082 33669285

[88] Fu H Kishore M Gittens B Wang G Coe D Komarowska I. Self-recognition of the endothelium enables regulatory T-cell trafficking and defines the kinetics of immune regulation. Nat Commun. (2014) 5:3436. doi: 10.1038/ncomms4436 24625653 PMC3959214

[89] Bäck M Yurdagul A Jr. Tabas I Öörni K Kovanen PT. Inflammation and its resolution in atherosclerosis: mediators and therapeutic opportunities. Nat Rev Cardiol. (2019) 16:389–406. doi: 10.1038/s41569-019-0169-2 30846875 PMC6727648

[90] Lawler PR Bhatt DL Godoy LC Lüscher TF Bonow RO Verma S. Targeting cardiovascular inflammation: next steps in clinical translation. Eur Heart J. (2021) 42:113–31. doi: 10.1093/eurheartj/ehaa099 32176778

[91] Kondapalli L Moslehi J Bonaca MP. Inflammation begets inflammation: cancer and acute MI. Eur Heart J. (2020) 41:2194–6. doi: 10.1093/eurheartj/ehz951 32068786

[92] Harb H Stephen-Victor E Crestani E Benamar M Massoud A Cui Y. A regulatory T cell Notch4-GDF15 axis licenses tissue inflammation in asthma. Nat Immunol. (2020) 21:1359–70. doi: 10.1038/s41590-020-0777-3 PMC7578174 32929274

[93] Sharabi A Tsokos MG Ding Y Malek TR Klatzmann D Tsokos GC. Regulatory T cells in the treatment of disease. Nat Rev Drug Discovery. (2018) 17:823–44. doi: 10.1038/nrd.2018.148 30310234

[94] Arpaia N Green JA Moltedo B Arvey A Hemmers S Yuan S. A distinct function of regulatory T cells in tissue protection. Cell. (2015) 162:1078–89. doi: 10.1016/j.cell.2015.08.021 PMC4603556 26317471

[95] Soehnlein O Libby P. Targeting inflammation in atherosclerosis - from experimental insights to the clinic. Nat Rev Drug Discovery. (2021) 20:589–610. doi: 10.1038/s41573-021-00198-1 33976384 PMC8112476

[96] Winkels H Meiler S Lievens D Engel D Spitz C Bürger C. CD27 co-stimulation increases the abundance of regulatory T cells and reduces atherosclerosis in hyperlipidaemic mice. Eur Heart J. (2017) 38:3590–9. doi: 10.1093/eurheartj/ehx517 29045618

[97] Wolf D Gerhardt T Winkels H Michel NA Pramod AB Ghosheh Y. Pathogenic autoimmunity in atherosclerosis evolves from initially protective apolipoprotein B(100)-reactive CD4(+) T-regulatory cells. Circulation. (2020) 142:1279–93. doi: 10.1161/CIRCULATIONAHA.119.042863 PMC7515473 32703007

[98] Lai M Peng H Wu X Chen X Wang B Su X. IL-38 in modulating hyperlipidemia and its related cardiovascular diseases. Int Immunopharmacol. (2022) 108:108876. doi: 10.1016/j.intimp.2022.108876 35623295

[99] Zhang H Ge S Ni B He K Zhu P Wu X. Augmenting ATG14 alleviates atherosclerosis and inhibits inflammation via promotion of autophagosome-lysosome fusion in macrophages. Autophagy. (2021) 17:4218–30. doi: 10.1080/15548627.2021.1909833 PMC8726734 33849389

[100] Bai Z Liu Y Zhao Y Yan R Yang L Ma H. Aspirin ameliorates atherosclerotic immuno-inflammation through regulating the Treg/Th17 axis and CD39-CD73 adenosine signaling via remodeling the gut microbiota in ApoE(-/-) mice. Int Immunopharmacol. (2023) 120:110296. doi: 10.1016/j.intimp.2023.110296 37187127

[101] Liao P Liu L Wang B Li W Fang X Guan S. Baicalin and geniposide attenuate atherosclerosis involving lipids regulation and immunoregulation in ApoE-/- mice. Eur J Pharmacol. (2014) 740:488–95. doi: 10.1016/j.ejphar.2014.06.039 24991786

[102] Fan Q Liu Y Rao J Zhang Z Xiao W Zhu T. Anti-atherosclerosis effect of Angong Niuhuang pill via regulating Th17/Treg immune balance and inhibiting chronic inflammatory on apoE(-/-) mice model of early and mid-term atherosclerosis. Front Pharmacol. (2019) 10:1584. doi: 10.3389/fphar.2019.01584 32082145 PMC7005527

[103] Wang F Liu M Ma D Cai Z Liu L Wang J. Dendritic cell-expressed IDO alleviates atherosclerosis by expanding CD4(+)CD25(+)Foxp3(+)Tregs through IDO-Kyn-AHR axis. Int Immunopharmacol. (2023) 116:109758. doi: 10.1016/j.intimp.2023.109758 36706593

[104] Tian Y Liang X Wu Y. The alternation of autophagy/apoptosis in CD4+CD25+Foxp3+ Tregs on the developmental stages of atherosclerosis. BioMed Pharmacother. (2018) 97:1053–60. doi: 10.1016/j.biopha.2017.11.013 29136784

[105] An T Guo M Fan C Huang S Liu H Liu K. sFgl2-Treg positive feedback pathway protects against atherosclerosis. Int J Mol Sci. (2023) 24(3):2338. doi: 10.3390/ijms24032338 36768661 PMC9916961

[106] Zhu J Fan J Xia Y Wang H Li Y Feng Z. Potential therapeutic targets of macrophages in inhibiting immune damage and fibrotic processes in musculoskeletal diseases. Front Immunol. (2023) 14:1219487. doi: 10.3389/fimmu.2023.1219487 37545490 PMC10400722

[107] Zhu L Jia L Liu Z Zhang Y Wang J Yuan Z. Elevated methylation of FOXP3 (Forkhead box P3)-TSDR (Regulatory T-cell-specific demethylated region) is associated with increased risk for adverse outcomes in patients with acute coronary syndrome. Hypertension. (2019) 74:581–9. doi: 10.1161/HYPERTENSIONAHA.119.12852 31327269

[108] Miyazaki T Taketomi Y Higashi T Ohtaki H Takaki T Ohnishi K. Hypercholesterolemic dysregulation of calpain in lymphatic endothelial cells interferes with regulatory T-cell stability and trafficking. Arterioscler Thromb Vasc Biol. (2023) 43:e66–82. doi: 10.1161/ATVBAHA.122.317781 36519468

[109] Ospina-Quintero L Jaramillo JC Tabares-Guevara JH Ramírez-Pineda JR. Reformulating small molecules for cardiovascular disease immune intervention: low-dose combined vitamin D/dexamethasone promotes IL-10 production and atheroprotection in dyslipidemic mice. Front Immunol. (2020) 11:743. doi: 10.3389/fimmu.2020.00743 32395119 PMC7197409

[110] Mailer RK Konrath S Zhan L Thode H Beerens M Frye M. Repetitive antigen responses of LDL-reactive CD4+ T cells induce Tr1 cell-mediated immune tolerance. Arterioscler Thromb Vasc Biol. (2023) 43:1510–23. doi: 10.1161/ATVBAHA.123.319135 37259863

[111] Pattarabanjird T Li C McNamara C. B cells in atherosclerosis: mechanisms and potential clinical applications. JACC Basic Transl Sci. (2021) 6:546–63. doi: 10.1016/j.jacbts.2021.01.006 PMC8246059 34222726

[112] Pattarabanjird T Marshall M Upadhye A Srikakulapu P Garmey JC Haider A. B-1b cells possess unique bHLH-driven P62-dependent self-renewal and atheroprotection. Circ Res. (2022) 130:981–93. doi: 10.1161/CIRCRESAHA.121.320436 PMC9075598 35209718

[113] Ma SD Mussbacher M Galkina EV. Functional role of B cells in atherosclerosis. Cells. (2021) 10(2):270. doi: 10.3390/cells10020270 33572939 PMC7911276

[114] Ketelhuth DF Hansson GK. Adaptive response of T and B cells in atherosclerosis. Circ Res. (2016) 118:668–78. doi: 10.1161/CIRCRESAHA.115.306427 26892965

[115] Mangge H Prüller F Schnedl W Renner W Almer G. Beyond macrophages and T cells: B cells and immunoglobulins determine the fate of the atherosclerotic plaque. Int J Mol Sci. (2020) 21(11):4082. doi: 10.3390/ijms21114082 32521607 PMC7312004

[116] Ait-Oufella H Sage AP Mallat Z Tedgui A. Adaptive (T and B cells) immunity and control by dendritic cells in atherosclerosis. Circ Res. (2014) 114:1640–60. doi: 10.1161/CIRCRESAHA.114.302761 24812352

[117] Walter K. What is acute myocarditis? Jama. (2023) 330:574. doi: 10.1001/jama.2023.5526 37552301

[118] Ammirati E Moslehi JJ. Diagnosis and treatment of acute myocarditis: A review. Jama. (2023) 329:1098–113. doi: 10.1001/jama.2023.3371 37014337

[119] Yu K Zhou L Wang Y Yu C Wang Z Liu H. Mechanisms and therapeutic strategies of viral myocarditis targeting autophagy. Front Pharmacol. (2022) 13:843103. doi: 10.3389/fphar.2022.843103 35479306 PMC9035591

[120] Liu X Li M Chen Z Yu Y Shi H Yu Y. Mitochondrial calpain-1 activates NLRP3 inflammasome by cleaving ATP5A1 and inducing mitochondrial ROS in CVB3-induced myocarditis. Basic Res Cardiol. (2022) 117:40. doi: 10.1007/s00395-022-00948-1 35997820 PMC9399059

[121] Shi H Yu Y Liu X Yu Y Li M Wang Y. Inhibition of calpain reduces cell apoptosis by suppressing mitochondrial fission in acute viral myocarditis. Cell Biol Toxicol. (2022) 38:487–504. doi: 10.1007/s10565-021-09634-9 34365571 PMC9200683

[122] Nadkarni R Chu WC Lee CQE Mohamud Y Yap L Toh GA. Viral proteases activate the CARD8 inflammasome in the human cardiovascular system. J Exp Med. (2022) 219(10):e20212117. doi: 10.1084/jem.20212117 36129453 PMC9499823

[123] Wang X Ge J Chen R. LAP(+) Treg is a better biomarker than total Treg in viral myocarditis. J Med Virol. (2019) 91:886–9. doi: 10.1002/jmv.25378 30570750

[124] Yan L Hu F Yan X Wei Y Ma W Wang Y. Inhibition of microRNA-155 ameliorates experimental autoimmune myocarditis by modulating Th17/Treg immune response. J Mol Med (Berl). (2016) 94:1063–79. doi: 10.1007/s00109-016-1414-3 27052830

[125] Lasrado N Borcherding N Arumugam R Starr TK Reddy J. Dissecting the cellular landscape and transcriptome network in viral myocarditis by single-cell RNA sequencing. iScience. (2022) 25:103865. doi: 10.1016/j.isci.2022.103865 35243228 PMC8861636

[126] Pappritz K Savvatis K Miteva K Kerim B Dong F Fechner H. Immunomodulation by adoptive regulatory T-cell transfer improves Coxsackievirus B3-induced myocarditis. FASEB J. (2018), fj201701408R. doi: 10.1096/fj.201701408R 29863913

[127] Wu J Liu M Mang G Yu S Chen Q Li T. Protosappanin A protects against experimental autoimmune myocarditis, and induces metabolically reprogrammed tolerogenic DCs. Pharmacol Res. (2019) 146:104269. doi: 10.1016/j.phrs.2019.104269 31078745

[128] Lee JH Kim TH Park HE Lee EG Jung NC Song JY. Myosin-primed tolerogenic dendritic cells ameliorate experimental autoimmune myocarditis. Cardiovasc Res. (2014) 101:203–10. doi: 10.1093/cvr/cvt246 24189626

[129] Vasconcelos JF Souza BS Lins TF Garcia LM Kaneto CM Sampaio GP. Administration of granulocyte colony-stimulating factor induces immunomodulation, recruitment of T regulatory cells, reduction of myocarditis and decrease of parasite load in a mouse model of chronic Chagas disease cardiomyopathy. FASEB J. (2013) 27:4691–702. doi: 10.1096/fj.13-229351 23964077

[130] Tajiri K Sakai S Kimura T Machino-Ohtsuka T Murakoshi N Xu D. Endothelin receptor antagonist exacerbates autoimmune myocarditis in mice. Life Sci. (2014) 118:288–96. doi: 10.1016/j.lfs.2014.01.007 24447632

[131] Heymans S Lakdawala NK Tschöpe C Klingel K. Dilated cardiomyopathy: causes, mechanisms, and current and future treatment approaches. Lancet. (2023) 402:998–1011. doi: 10.1016/S0140-6736(23)01241-2 37716772

[132] Lakdawala NK Winterfield JR Funke BH. Dilated cardiomyopathy. Nat Rev Dis Primers. (2019) 5:33. doi: 10.1038/s41572-019-0088-x 31073134

[133] Santos ES de Aragão-França LS Meira CS Cerqueira JV Vasconcelos JF Nonaka CKV. Tolerogenic dendritic cells reduce cardiac inflammation and fibrosis in chronic Chagas disease. Front Immunol. (2020) 11:488. doi: 10.3389/fimmu.2020.00488 32318058 PMC7154094

[134] Verdonschot JAJ Cooper LT Heymans SRB. Parvovirus B19 in dilated cardiomyopathy: there is more than meets the eye. J Card Fail. (2019) 25:64–6. doi: 10.1016/j.cardfail.2018.11.017 30481561

[135] Ge T Yu Y Cui J Cai L. The adaptive immune role of metallothioneins in the pathogenesis of diabetic cardiomyopathy: good or bad. Am J Physiol Heart Circ Physiol. (2019) 317:H264–h275. doi: 10.1152/ajpheart.00123.2019 31100011

[136] Wei J Li B Wang X Li X Hu Y Qiao L. Efficacy and safety of Qili Qiangxin capsule on dilated cardiomyopathy: A systematic review and meta-analysis of 35 randomized controlled trials. Front Pharmacol. (2022) 13:893602. doi: 10.3389/fphar.2022.893602 35571117 PMC9095857

[137] Nunes JPS Andrieux P Brochet P Almeida RR Kitano E Honda AK. Co-exposure of cardiomyocytes to IFN-γ and TNF-α Induces mitochondrial dysfunction and nitro-oxidative stress: implications for the pathogenesis of chronic Chagas disease cardiomyopathy. Front Immunol. (2021) 12:755862. doi: 10.3389/fimmu.2021.755862 34867992 PMC8632642

[138] Gong C Chang L Sun X Qi Y Huang R Chen K. Infusion of two-dose mesenchymal stem cells is more effective than a single dose in a dilated cardiomyopathy rat model by upregulating indoleamine 2,3-dioxygenase expression. Stem Cell Res Ther. (2022) 13:409. doi: 10.1186/s13287-022-03101-w 35962420 PMC9373305

[139] Zhu ZF Tang TT Dong WY Li YY Xia N Zhang WC. Defective circulating CD4+LAP+ regulatory T cells in patients with dilated cardiomyopathy. J Leukoc Biol. (2015) 97:797–805. doi: 10.1189/jlb.5A1014-469RR 25722319

[140] Wei Y Yu K Wei H Su X Zhu R Shi H. CD4(+) CD25(+) GARP(+) regulatory T cells display a compromised suppressive function in patients with dilated cardiomyopathy. Immunology. (2017) 151:291–303. doi: 10.1111/imm.12728 28207945 PMC5461097

[141] Yin H Guo X Chen Y Zeng Y Mo X Hong S. TAB2 deficiency induces dilated cardiomyopathy by promoting RIPK1-dependent apoptosis and necroptosis. J Clin Invest. (2022) 132(4):e152297. doi: 10.1172/JCI152297 34990405 PMC8843707

[142] Czepiel M Diviani D Jaźwa-Kusior A Tkacz K Rolski F Smolenski RT. Angiotensin II receptor 1 controls profibrotic Wnt/β-catenin signalling in experimental autoimmune myocarditis. Cardiovasc Res. (2022) 118:573–84. doi: 10.1093/cvr/cvab039 PMC8803091 33576779

[143] Gast M Rauch BH Haghikia A Nakagawa S Haas J Stroux A. Long noncoding RNA NEAT1 modulates immune cell functions and is suppressed in early onset myocardial infarction patients. Cardiovasc Res. (2019) 115:1886–906. doi: 10.1093/cvr/cvz085 30924864

[144] Blanton RM Carrillo-Salinas FJ Alcaide P. T-cell recruitment to the heart: friendly guests or unwelcome visitors? Am J Physiol Heart Circ Physiol. (2019) 317:H124–h140. doi: 10.1152/ajpheart.00028.2019 31074651 PMC6692732

[145] Misra S Shishehbor MH Takahashi EA Aronow HD Brewster LP Bunte MC. Perfusion assessment in critical limb ischemia: principles for understanding and the development of evidence and evaluation of devices: A scientific statement from the American Heart Association. Circulation. (2019) 140:e657–72. doi: 10.1161/CIR.0000000000000708 PMC7372288 31401843

[146] Wang Y Dembowsky K Chevalier E Stüve P Korf-Klingebiel M Lochner M. C-X-C motif chemokine receptor 4 blockade promotes tissue repair after myocardial infarction by enhancing regulatory T cell mobilization and immune-regulatory function. Circulation. (2019) 139:1798–812. doi: 10.1161/CIRCULATIONAHA.118.036053 PMC6467561 30696265

[147] Ahamad N Kar A Mehta S Dewani M Ravichandran V Bhardwaj P. Immunomodulatory nanosystems for treating inflammatory diseases. Biomaterials. (2021) 274:120875. doi: 10.1016/j.biomaterials.2021.120875 34010755

[148] Sun K Li YY Jin J. A double-edged sword of immuno-microenvironment in cardiac homeostasis and injury repair. Signal Transduct Target Ther. (2021) 6:79. doi: 10.1038/s41392-020-00455-6 33612829 PMC7897720

[149] Zeng Z Yu K Chen L Li W Xiao H Huang Z. Interleukin-2/Anti-Interleukin-2 Immune Complex Attenuates Cardiac Remodeling after Myocardial Infarction through Expansion of Regulatory T Cells. J Immunol Res. (2016) 2016:8493767. doi: 10.1155/2016/8493767 27144181 PMC4837274

[150] Zhang Y Cai Z Shen Y Lu Q Gao W Zhong X. Hydrogel-load exosomes derived from dendritic cells improve cardiac function via Treg cells and the polarization of macrophages following myocardial infarction. J Nanobiotechnol. (2021) 19:271. doi: 10.1186/s12951-021-01016-x PMC8424987 34496871

[151] Zhang M Zhang S. T cells in fibrosis and fibrotic diseases. Front Immunol. (2020) 11:1142. doi: 10.3389/fimmu.2020.01142 32676074 PMC7333347

[152] Sun X Feng Y Gong C Bao X Wei Z Chang L. Hypertension-driven regulatory T-cell perturbations accelerate myocardial ischemia-reperfusion injury. Hypertension. (2023) 80:2046–58. doi: 10.1161/HYPERTENSIONAHA.123.20481 37615092

[153] Feng G Bajpai G Ma P Koenig A Bredemeyer A Lokshina I. CCL17 aggravates myocardial injury by suppressing recruitment of regulatory T cells. Circulation. (2022) 145:765–82. doi: 10.1161/CIRCULATIONAHA.121.055888 PMC8957788 35113652

[154] Weirather J Hofmann UD Beyersdorf N Ramos GC Vogel B Frey A. Foxp3+ CD4+ T cells improve healing after myocardial infarction by modulating monocyte/macrophage differentiation. Circ Res. (2014) 115:55–67. doi: 10.1161/CIRCRESAHA.115.303895 24786398

[155] Delgobo M Weiß E Ashour D Richter L Popiolkowski L Arampatzi P. Myocardial milieu favors local differentiation of regulatory T cells. Circ Res. (2023) 132:565–82. doi: 10.1161/CIRCRESAHA.122.322183 36744467

[156] Weiß E Ramos GC Delgobo M. Myocardial-Treg crosstalk: how to tame a wolf. Front Immunol. (2022) 13:914033. doi: 10.3389/fimmu.2022.914033 35693830 PMC9176752

[157] Sharir R Semo J Shimoni S Ben-Mordechai T Landa-Rouben N Maysel-Auslender S. Experimental myocardial infarction induces altered regulatory T cell hemostasis, and adoptive transfer attenuates subsequent remodeling. PloS One. (2014) 9:e113653. doi: 10.1371/journal.pone.0113653 25436994 PMC4249913

[158] Bezerra OC França CM Rocha JA Neves GA Souza PRM Teixeira Gomes M. Cholinergic stimulation improves oxidative stress and inflammation in experimental myocardial infarction. Sci Rep. (2017) 7:13687. doi: 10.1038/s41598-017-14021-8 29057895 PMC5651932

[159] Karthikeyan G Guilherme L. Acute rheumatic fever. Lancet. (2018) 392:161–74. doi: 10.1016/S0140-6736(18)30999-1 30025809

[160] Remenyi B ElGuindy A Smith SC Jr. Yacoub M Holmes DR Jr. Valvular aspects of rheumatic heart disease. Lancet. (2016) 387:1335–46. doi: 10.1016/S0140-6736(16)00547-X 27025439

[161] Marijon E Celermajer DS Jouven X. Rheumatic heart disease - an iceberg in tropical waters. N Engl J Med. (2017) 377:780–1. doi: 10.1056/NEJMe1705840 28834468

[162] Coffey S Roberts-Thomson R Brown A Carapetis J Chen M Enriquez-Sarano M. Global epidemiology of valvular heart disease. Nat Rev Cardiol. (2021) 18:853–64. doi: 10.1038/s41569-021-00570-z 34172950

[163] Mukhopadhyay S Varma S Mohan Kumar HN Yusaf J Goyal M Mehta V. Circulating level of regulatory T cells in rheumatic heart disease: An observational study. Indian Heart J. (2016) 68:342–8. doi: 10.1016/j.ihj.2015.08.009 PMC4911432 27316488

[164] Bas HD Baser K Yavuz E Bolayir HA Yaman B Unlu S. A shift in the balance of regulatory T and T helper 17 cells in rheumatic heart disease. J Investig Med. (2014) 62:78–83. doi: 10.2310/JIM.0000000000000023 24158043

[165] Toor D Sharma N. T cell subsets: an integral component in pathogenesis of rheumatic heart disease. Immunol Res. (2018) 66:18–30. doi: 10.1007/s12026-017-8978-z 29170852

[166] Jiang T Zhao Q Sun H Zhang L Song S Chi H. Scolopendra subspinipes mutilans L. Koch Ameliorates Rheumatic Heart Disease by Affecting Relative Percentages of CD4(+)CD25(+)FoxP3 Treg and CD4(+)IL17 T Cells. Evid Based Complement Alternat Med. (2019) 2019:4674190. doi: 10.1155/2019/4674190 31379962 PMC6662451

[167] Mukhopadhyay S Varma S Gade S Yusuf J Trehan V Tyagi S. Regulatory T-cell deficiency in rheumatic heart disease: a preliminary observational study. J Heart Valve Dis. (2013) 22:118–25. 23610999

[168] Rizzoni D De Ciuceis C Szczepaniak P Paradis P Schiffrin EL Guzik TJ. Immune system and microvascular remodeling in humans. Hypertension. (2022) 79:691–705. doi: 10.1161/HYPERTENSIONAHA.121.17955 35098718

[169] Cui C Fan J Zeng Q Cai J Chen Y Chen Z. CD4(+) T-cell endogenous cystathionine γ Lyase-hydrogen sulfide attenuates hypertension by sulfhydrating liver kinase B1 to promote T regulatory cell differentiation and proliferation. Circulation. (2020) 142:1752–69. doi: 10.1161/CIRCULATIONAHA.119.045344 32900241

[170] Gray G Scroggins DG Wilson KT Scroggins SM. Cellular immunotherapy in mice prevents maternal hypertension and restores anti-inflammatory cytokine balance in maternal and fetal tissues. Int J Mol Sci. (2023) 24(17):13594. doi: 10.1101/2023.08.14.553300 37686399 PMC10487605

[171] Tian W Jiang SY Jiang X Tamosiuniene R Kim D Guan T. The role of regulatory T cells in pulmonary arterial hypertension. Front Immunol. (2021) 12:684657. doi: 10.3389/fimmu.2021.684657 34489935 PMC8418274

[172] Zhang J Crowley SD. Role of T lymphocytes in hypertension. Curr Opin Pharmacol. (2015) 21:14–9. doi: 10.1016/j.coph.2014.12.003 PMC4380788 25523165

[173] Failer T Amponsah-Offeh M Neuwirth A Kourtzelis I Subramanian P Mirtschink P. Developmental endothelial locus-1 protects from hypertension-induced cardiovascular remodeling via immunomodulation. J Clin Invest. (2022) 474(9):963–4. doi: 10.1172/JCI126155 PMC11060724 38690740

[174] Ren J Crowley SD. Role of T-cell activation in salt-sensitive hypertension. Am J Physiol Heart Circ Physiol. (2019) 316:H1345–h1353. doi: 10.1152/ajpheart.00096.2019 30901277 PMC6620682

[175] Rudemiller NP Crowley SD. The role of chemokines in hypertension and consequent target organ damage. Pharmacol Res. (2017) 119:404–11. doi: 10.1016/j.phrs.2017.02.026 PMC5424532 28279813

[176] Schiffrin EL. Immune mechanisms in hypertension and vascular injury. Clin Sci (Lond). (2014) 126:267–74. doi: 10.1042/CS20130407 24144355

[177] Cornelius DC Cottrell J Amaral LM LaMarca B. Inflammatory mediators: a causal link to hypertension during preeclampsia. Br J Pharmacol. (2019) 176:1914–21. doi: 10.1111/bph.14466 PMC6534812 30095157

[178] Chen CN Hajji N Yeh FC Rahman S Ali S Wharton J. Restoration of Foxp3(+) regulatory T cells by HDAC-dependent epigenetic modulation plays a pivotal role in resolving pulmonary arterial hypertension pathology. Am J Respir Crit Care Med. (2023) 208:879–95. doi: 10.1164/rccm.202301-0181OC 37676930

[179] Kasal DA Barhoumi T Li MW Yamamoto N Zdanovich E Rehman A. T regulatory lymphocytes prevent aldosterone-induced vascular injury. Hypertension. (2012) 59:324–30. doi: 10.1161/HYPERTENSIONAHA.111.181123 22146512

[180] Barhoumi T Kasal DA Li MW Shbat L Laurant P Neves MF. T regulatory lymphocytes prevent angiotensin II-induced hypertension and vascular injury. Hypertension. (2011) 57:469–76. doi: 10.1161/HYPERTENSIONAHA.110.162941 21263125

[181] Iulita MF Duchemin S Vallerand D Barhoumi T Alvarez F Istomine R. CD4(+) regulatory T lymphocytes prevent impaired cerebral blood flow in angiotensin II-induced hypertension. J Am Heart Assoc. (2019) 8:e009372. doi: 10.1161/JAHA.118.009372 30572753 PMC6405729

[182] Pollow DP Jr. Uhlorn JA Sylvester MA Romero-Aleshire MJ Uhrlaub JL Lindsey ML. Menopause and FOXP3(+) Treg cell depletion eliminate female protection against T cell-mediated angiotensin II hypertension. Am J Physiol Heart Circ Physiol. (2019) 317:H415–h423. doi: 10.1152/ajpheart.00792.2018 31099612 PMC6732479

[183] Cicco S Desantis V Vacca A Cazzato G Solimando AG Cirulli A. Cardiovascular Risk in Patients With Takayasu Arteritis Directly Correlates With Diastolic Dysfunction and Inflammatory Cell Infiltration in the Vessel Wall: A Clinical, ex vivo and in *vitro* Analysis. Front Med (Lausanne). (2022) 9:863150. doi: 10.3389/fmed.2022.863150 35652080 PMC9149422

[184] Klingenberg R Brokopp CE Grivès A Courtier A Jaguszewski M Pasqual N. Clonal restriction and predominance of regulatory T cells in coronary thrombi of patients with acute coronary syndromes. Eur Heart J. (2015) 36:1041–8. doi: 10.1093/eurheartj/eht543 PMC4416137 24419807

[185] Radwan E Mali V Haddox S El-Noweihi A Mandour M Ren J. Treg cells depletion is a mechanism that drives microvascular dysfunction in mice with established hypertension. Biochim Biophys Acta Mol Basis Dis. (2019) 1865:403–12. doi: 10.1016/j.bbadis.2018.10.031 30414897

[186] Oh S Yang JY Park CH Son KH Byun K. Dieckol reduces muscle atrophy by modulating angiotensin type II type 1 receptor and NADPH oxidase in spontaneously hypertensive rats. Antioxid (Basel). (2021) 10(10):1561. doi: 10.3390/antiox10101561 PMC8533257 34679696

[187] Tamosiuniene R Manouvakhova O Mesange P Saito T Qian J Sanyal M. Dominant role for regulatory T cells in protecting females against pulmonary hypertension. Circ Res. (2018) 122:1689–702. doi: 10.1161/CIRCRESAHA.117.312058 PMC5993601 29545367

[188] Kvakan H Kleinewietfeld M Qadri F Park JK Fischer R Schwarz I. Regulatory T cells ameliorate angiotensin II-induced cardiac damage. Circulation. (2009) 119:2904–12. doi: 10.1161/CIRCULATIONAHA.108.832782 19470887

[189] Martins S António N Carvalheiro T Laranjeira P Rodrigues R Gonçalves L. Reduced numbers of regulatory T cells in chronic heart failure seems not to be restored by cardiac resynchronization therapy. BMC Cardiovasc Disord. (2023) 23:89. doi: 10.1186/s12872-023-03109-x 36792985 PMC9933267

[190] Ovchinnikov A Filatova A Potekhina A Arefieva T Gvozdeva A Ageev F. Blood immune cell alterations in patients with hypertensive left ventricular hypertrophy and heart failure with preserved ejection fraction. J Cardiovasc Dev Dis. (2023) 10(7):310. doi: 10.3390/jcdd10070310 37504566 PMC10380876

[191] Li N Bian H Zhang J Li X Ji X Zhang Y. The Th17/Treg imbalance exists in patients with heart failure with normal ejection fraction and heart failure with reduced ejection fraction. Clin Chim Acta. (2010) 411:1963–8. doi: 10.1016/j.cca.2010.08.013 20713031

[192] Zhang Q Hu LQ Yin CS Chen P Li HQ Sun X. Catechin ameliorates cardiac dysfunction in rats with chronic heart failure by regulating the balance between Th17 and Treg cells. Inflammation Res. (2014) 63:619–28. doi: 10.1007/s00011-014-0734-4 24760105

[193] Liao S Tang Y Yue X Gao R Yao W Zhou Y. β-hydroxybutyrate mitigated heart failure with preserved ejection fraction by increasing Treg cells via Nox2/GSK-3β. J Inflammation Res. (2021) 14:4697–706. doi: 10.2147/JIR.S331320 PMC8453303 34557014

[194] Bansal SS Ismahil MA Goel M Patel B Hamid T Rokosh G. Activated T lymphocytes are essential drivers of pathological remodeling in ischemic heart failure. Circ Heart Fail. (2017) 10:e003688. doi: 10.1161/CIRCHEARTFAILURE.116.003688 28242779 PMC5331621

[195] Alvarez F Istomine R Shourian M Pavey N Al-Aubodah TA Qureshi S. The alarmins IL-1 and IL-33 differentially regulate the functional specialisation of Foxp3(+) regulatory T cells during mucosal inflammation. Mucosal Immunol. (2019) 12:746–60. doi: 10.1038/s41385-019-0153-5 30872761

[196] Xue Z Zhang X Chen M Lu X Deng R Ma Y. Dendritic cells transduced with single immunoglobulin IL-1-related receptor exhibit immature properties and prolong islet allograft survival. Front Immunol. (2017) 8:1671. doi: 10.3389/fimmu.2017.01671 29250066 PMC5714859

[197] Ridker PM Everett BM Thuren T MacFadyen JG Chang WH Ballantyne C. Antiinflammatory therapy with canakinumab for atherosclerotic disease. N sEngl J Med. (2017) 377:1119–31. doi: 10.1056/NEJMoa1707914 28845751

[198] Ritvo PG Churlaud G Quiniou V Florez L Brimaud F Fourcade G. T(fr) cells lack IL-2Rα but express decoy IL-1R2 and IL-1Ra and suppress the IL-1-dependent activation of T(fh) cells. Sci Immunol. (2017) 2(15):eaan0368. doi: 10.1126/sciimmunol.aan0368 28887367

[199] Graßhoff H Comdühr S Monne LR Müller A Lamprecht P Riemekasten G. Low-dose IL-2 therapy in autoimmune and rheumatic diseases. Front Immunol. (2021) 12:648408. doi: 10.3389/fimmu.2021.648408 33868284 PMC8047324

[200] Zhao TX Kostapanos M Griffiths C Arbon EL Hubsch A Kaloyirou F. Low-dose interleukin-2 in patients with stable ischaemic heart disease and acute coronary syndromes (LILACS): protocol and study rationale for a randomised, double-blind, placebo-controlled, phase I/II clinical trial. BMJ Open. (2018) 8:e022452. doi: 10.1136/bmjopen-2018-022452 PMC6144322 30224390

[201] Khoryati L Pham MN Sherve M Kumari S Cook K Pearson J. An IL-2 mutein engineered to promote expansion of regulatory T cells arrests ongoing autoimmunity in mice. Sci Immunol. (2020) 5(50):eaba5264. doi: 10.1126/sciimmunol.aba5264 32817295 PMC7643170

[202] Sniderman AD Thanassoulis G Glavinovic T Navar AM Pencina M Catapano A. Apolipoprotein B particles and cardiovascular disease: A narrative review. JAMA Cardiol. (2019) 4:1287–95. doi: 10.1001/jamacardio.2019.3780 PMC7369156 31642874

[203] Muscella A Stefàno E Marsigliante S. The effects of exercise training on lipid metabolism and coronary heart disease. Am J Physiol Heart Circ Physiol. (2020) 319:H76–h88. doi: 10.1152/ajpheart.00708.2019 32442027

[204] Marston NA Giugliano RP Melloni GEM Park JG Morrill V Blazing MA. Association of apolipoprotein B-containing lipoproteins and risk of myocardial infarction in individuals with and without atherosclerosis: distinguishing between particle concentration, type, and content. JAMA Cardiol. (2022) 7:250–6. doi: 10.1001/jamacardio.2021.5083 PMC8590731 34773460

[205] Yi S Zhang X Sangji H Liu Y Allen SD Xiao B. Surface engineered polymersomes for enhanced modulation of dendritic cells during cardiovascular immunotherapy. Adv Funct Mater. (2019) 29(42):1904399. doi: 10.1002/adfm.201904399 34335131 PMC8320590

[206] Yuan S Tang B Zheng J Larsson SC. Circulating lipoprotein lipids, apolipoproteins and ischemic stroke. Ann Neurol. (2020) 88:1229–36. doi: 10.1002/ana.25916 PMC7756401 32981134

[207] Hagström E Steg PG Szarek M Bhatt DL Bittner VA Danchin N. Apolipoprotein B, residual cardiovascular risk after acute coronary syndrome, and effects of alirocumab. Circulation. (2022) 146:657–72. doi: 10.1161/CIRCULATIONAHA.121.057807 PMC9422774 35770629

[208] Lehrer-Graiwer J Singh P Abdelbaky A Vucic E Korsgren M Baruch A. FDG-PET imaging for oxidized LDL in stable atherosclerotic disease: a phase II study of safety, tolerability, and anti-inflammatory activity. JACC Cardiovasc Imaging. (2015) 8:493–4. doi: 10.1016/j.jcmg.2014.06.021 25457756

[209] Marangoni F Zhakyp A Corsini M Geels SN Carrizosa E Thelen M. Expansion of tumor-associated Treg cells upon disruption of a CTLA-4-dependent feedback loop. Cell. (2021) 184:3998–4015.e19. doi: 10.1016/j.cell.2021.05.027 34157302 PMC8664158

[210] Madonna G Ballesteros-Merino C Feng Z Bifulco C Capone M Giannarelli D. PD-L1 expression with immune-infiltrate evaluation and outcome prediction in melanoma patients treated with ipilimumab. Oncoimmunology. (2018) 7:e1405206. doi: 10.1080/2162402X.2017.1405206 30524879 PMC6279420

[211] Amoozgar Z Kloepper J Ren J Tay RE Kazer SW Kiner E. Targeting Treg cells with GITR activation alleviates resistance to immunotherapy in murine glioblastomas. Nat Commun. (2021) 12:2582. doi: 10.1038/s41467-021-22885-8 33976133 PMC8113440

[212] Sabatine MS Giugliano RP Keech AC Honarpour N Wiviott SD Murphy SA. Evolocumab and clinical outcomes in patients with cardiovascular disease. N Engl J Med. (2017) 376:1713–22. doi: 10.1056/NEJMoa1615664 28304224

[213] Spitz C Winkels H Bürger C Weber C Lutgens E Hansson GK. Regulatory T cells in atherosclerosis: critical immune regulatory function and therapeutic potential. Cell Mol Life Sci. (2016) 73:901–22. doi: 10.1007/s00018-015-2080-2 PMC11108393 26518635

[214] Li Q Wang Y Li H Shen G Hu S. Ox-LDL influences peripheral Th17/Treg balance by modulating Treg apoptosis and Th17 proliferation in atherosclerotic cerebral infarction. Cell Physiol Biochem. (2014) 33:1849–62. doi: 10.1159/000362963 24969587

[215] Beers DR Thonhoff JR Faridar A Thome AD Zhao W Wen S. Tregs attenuate peripheral oxidative stress and acute phase proteins in ALS. Ann Neurol. (2022) 92:195–200. doi: 10.1002/ana.26375 35445431 PMC9545429

[216] Momtazi-Borojeni AA Jaafari MR Afshar M Banach M Sahebkar A. PCSK9 immunization using nanoliposomes: preventive efficacy against hypercholesterolemia and atherosclerosis. Arch Med Sci. (2021) 17:1365–77. doi: 10.5114/aoms/133885 PMC8425258 34522266

[217] Momtazi-Borojeni AA Jaafari MR Banach M Gorabi AM Sahraei H Sahebkar A. Pre-clinical evaluation of the nanoliposomal antiPCSK9 vaccine in healthy non-human primates. Vaccines (Basel). (2021) 9(7):749. doi: 10.3390/vaccines9070749 34358164 PMC8309966

[218] Momtazi-Borojeni AA Jaafari MR Badiee A Sahebkar A. Long-term generation of antiPCSK9 antibody using a nanoliposome-based vaccine delivery system. Atherosclerosis. (2019) 283:69–78. doi: 10.1016/j.atherosclerosis.2019.02.001 30797988

[219] Zeitlinger M Bauer M Reindl-Schwaighofer R Stoekenbroek RM Lambert G Berger-Sieczkowski E. A phase I study assessing the safety, tolerability, immunogenicity, and low-density lipoprotein cholesterol-lowering activity of immunotherapeutics targeting PCSK9. Eur J Clin Pharmacol. (2021) 77:1473–84. doi: 10.1007/s00228-021-03149-2 PMC8440313 33969434

[220] Song X Sun X Oh SF Wu M Zhang Y Zheng W. Microbial bile acid metabolites modulate gut RORγ(+) regulatory T cell homeostasis. Nature. (2020) 577:410–5. doi: 10.1038/s41586-019-1865-0 PMC7274525 31875848

[221] Calcaterra V Croce S Vinci F De Silvestri A Cordaro E Regalbuto C. Th17 and Treg balance in children with obesity and metabolically altered status. Front Pediatr. (2020) 8:591012. doi: 10.3389/fped.2020.591012 33330284 PMC7710792

[222] Huber SA Feldman AM Sartini D. Coxsackievirus B3 induces T regulatory cells, which inhibit cardiomyopathy in tumor necrosis factor-alpha transgenic mice. Circ Res. (2006) 99:1109–16. doi: 10.1161/01.RES.0000249405.13536.49 17038643

[223] Bracamonte-Baran W Čiháková D. Cardiac autoimmunity: myocarditis. Adv Exp Med Biol. (2017) 1003:187–221. doi: 10.1007/978-3-319-57613-8_10 28667560 PMC5706653

[224] Saigusa R Winkels H Ley K. T cell subsets and functions in atherosclerosis. Nat Rev Cardiol. (2020) 17:387–401. doi: 10.1038/s41569-020-0352-5 32203286 PMC7872210

[225] Meng X Yang J Dong M Zhang K Tu E Gao Q. Regulatory T cells in cardiovascular diseases. Nat Rev Cardiol. (2016) 13:167–79. doi: 10.1038/nrcardio.2015.169 PMC11849084 26525543

[226] Scheinecker C Göschl L Bonelli M. Treg cells in health and autoimmune diseases: New insights from single cell analysis. J Autoimmun. (2020) 110:102376. doi: 10.1016/j.jaut.2019.102376 31862128

[227] Savage PA Klawon DEJ Miller CH. Regulatory T cell development. Annu Rev Immunol. (2020) 38:421–53. doi: 10.1146/annurev-immunol-100219-020937 31990619

[228] Raffin C Vo LT Bluestone JA. T(reg) cell-based therapies: challenges and perspectives. Nat Rev Immunol. (2020) 20:158–72. doi: 10.1038/s41577-019-0232-6 PMC7814338 31811270

[229] Hanna BS Wang G Galván-Peña S Mann AO Ramirez RN Muñoz-Rojas AR. The gut microbiota promotes distal tissue regeneration via RORγ(+) regulatory T cell emissaries. Immunity. (2023) 56:829–846.e8. doi: 10.1016/j.immuni.2023.01.033 36822206 PMC10101925

